# Conjunctival fibrosis and the innate barriers to Chlamydia trachomatis intracellular infection: a genome wide association study

**DOI:** 10.1038/srep17447

**Published:** 2015-11-30

**Authors:** Chrissy h. Roberts, Christopher S. Franklin, Pateh Makalo, Hassan Joof, Isatou Sarr, Olaimatu S. Mahdi, Ansumana Sillah, Momodou Bah, Felicity Payne, Anna E. Jeffreys, William Bottomley, Angels Natividad, Sandra Molina-Gonzalez, Sarah E. Burr, Mark Preston, Dominic Kwiatkowski, Kirk A. Rockett, Taane G. Clark, Matthew J. Burton, David C. W. Mabey, Robin Bailey, Inês Barroso, Martin J. Holland

**Affiliations:** 1 London School of Hygiene and Tropical Medicine, London, UK; 2 Wellcome Trust Sanger Institute, Hinxton, UK; 3 Medical Research Council Unit, The Gambia, Atlantic Boulevard, Fajara, The Gambia; 4 National Eye Care Programme, Gambian Ministry of Health, Banjul, The Gambia; 5 Sightsavers International, The Gambia. Kairaba Avenue, Banjul, The Gambia; 6 International Centre for Eye Health, London, UK; 7 Wellcome Trust Centre for Human Genetics, Oxford, UK; 8 University of Cambridge Metabolic Research Laboratories, Wellcome Trust-MRC Institute of Metabolic Science, Cambridge, UK; 9 NIHR Cambridge Biomedical Research Centre, Cambridge, UK

## Abstract

*Chlamydia trachomatis* causes both trachoma and sexually transmitted infections. These diseases have similar pathology and potentially similar genetic predisposing factors. We aimed to identify polymorphisms and pathways associated with pathological sequelae of ocular *Chlamydia trachomatis* infections in The Gambia. We report a discovery phase genome-wide association study (GWAS) of scarring trachoma (1090 cases, 1531 controls) that identified 27 SNPs with strong, but not genome-wide significant, association with disease (5 × 10^−6^ > P > 5 × 10^−8^). The most strongly associated SNP (rs111513399, P = 5.38 × 10^−7^) fell within a gene (*PREX2*) with homology to factors known to facilitate chlamydial entry to the host cell. Pathway analysis of GWAS data was significantly enriched for mitotic cell cycle processes (P = 0.001), the immune response (P = 0.00001) and for multiple cell surface receptor signalling pathways. New analyses of published transcriptome data sets from Gambia, Tanzania and Ethiopia also revealed that the same cell cycle and immune response pathways were enriched at the transcriptional level in various disease states. Although unconfirmed, the data suggest that genetic associations with chlamydial scarring disease may be focussed on processes relating to the immune response, the host cell cycle and cell surface receptor signalling.

*Chlamydia trachomatis* (Ct) infections have a significant impact on global health. As the cause of trachoma, Ct is the most common infectious cause of blindness^1^. The same bacterium is attributed to 106 million sexually transmitted infections (STIs) per annum^2^. Chronic immunopathological reactions have been implicated as the primary cause of pathology resulting from Ct infections^3^ and in both trachoma and STIs the primary pathological mechanism is the progressive formation of fibrotic scars at the site of infection^4^,^5^,^6^. In STIs, this can ultimately lead to infection related infertility and ectopic pregnancy^7^, whilst in the eye, trachomatous scarring (TS) causes the tarsal plate of the eyelid to become deformed, leading to entropion and trachomatous trichiasis (TT). When TT occurs, the eyelashes turn towards the globe of the eye and scratch the cornea, causing pain, opacity, visual impairment and blindness. Since the TS phenotype can be readily observed in the field using a magnifying loupe, trachoma represents an ideal platform for the study of genetic and biological factors that modulate the pathophysiological response to human Ct infections. The study of pathology in the reproductive tract meanwhile requires invasive techniques and it is difficult to obtain specimens from large numbers of confirmed Ct-STI-related scarring cases.

Not all individuals who become infected with Ct (either through STI or in the eye) will suffer clinically significant levels of disease. Those who suffer repeated inflammatory episodes at the conjunctiva have been identified as being at high risk of progressing to TS^8^ and a similarly increased risk of genitourinary tract pathology has been linked to repeated Ct STIs^6^. The observation of TS correlates well with increased inflammatory cell activity in the epithelium as measured using *in vivo* confocal microscopy^9^. Substantial amounts of pathology can be attributed to environmental risk factors, but the familial clustering^10^ of TS, the transmission disequilibrium of TS risk alleles^11^ and evidence from studies focussed on immune response genes^12^,^13^,^14^,^15^,^16^,^17^,^18^,^19^ each point to a significant role for host genetics in determining risk of developing TS.

Infectious diseases have been relatively less studied by genome-wide association study (GWAS) than non-communicable diseases. Surprisingly few genome-wide associations were reported in several large-scale GWAS^20^,^21^,^22^ and this could be attributed to the reduced power of the studies which resulted from the increased genetic diversity, complex population structures and the low degree of linkage disequilibrium (LD) in the studied populations^23^. Recent developments in pathways based analysis of GWAS data enable more extensive, multi-SNP analysis and the collapsing of information across networks of functionally interacting polymorphisms^24^,^25^,^26^,^27^. This may add substantially to the discovery power of GWAS in complex phenotypes such as infectious diseases^28^.

The aim of this work was identify candidate human SNPs, loci and pathways that are associated with protection or predisposition of the host to pathological sequelae of ocular Ct infections. To do this, we tested the association of ~1.5 million directly genotyped and ~9 million imputed SNPs with disease status in 1090 TS cases and 1531 controls from The Gambia. Using the same GWAS genotyping data we went on to use Pathways of Distinction Analysis (PODA)^25^ and the Assignment List GO AnnotaTOR (ALIGATOR)^26^ to test the association between TS and 1345 multi-gene pathways. We finally sought to support the GWAS pathway level analysis features by performing a pathways level analysis on Gene Expression Omnibus (GEO) deposited trachoma data-sets from The Gambia, Tanzania and Ethiopia.

## Results

### Sample Demographics

**Figure 1:**
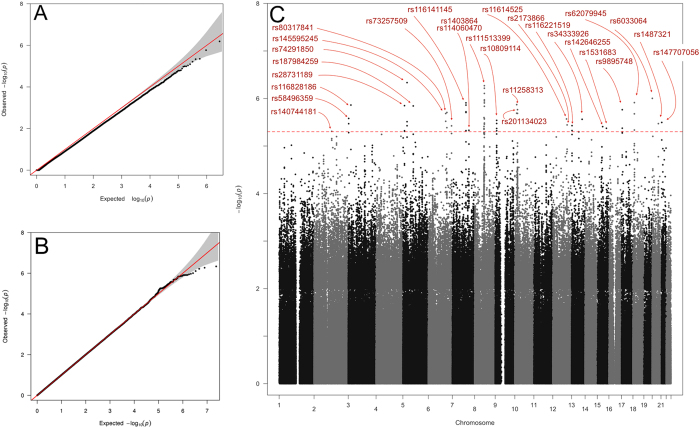
Results of GWAS Analysis using EMMAX. (**A**) QQ plot: There is genome-wide deflation (λ = 0.982 SE = 1.74e^−05^) of the test statistic when the phenotype is corrected for kinship only. (**B**) QQ plot: Phenotype correction for age, gender and pairwise kinship removed any deviation from the null expectation of the genome-wide test statistic (λ = 1.001, SE = 8.7e^−7^). (**C**) Manhattan plot showing index SNPs with P_EMMAX_ < 5 × 10^−6^.

The case and control groups were approximately equivalent with respect to gender and ethnicity. 70% of cases and 63% of controls were female. Self-described ethnic composition of the cases was 29% Jola, 27% Mandinka, 21% Wolof, 10% Fula and 13% other/no data. In controls the composition was 25% Jola, 24% Mandinka, 21% Wolof, 8% Fula and 22% other/no data. Median age was 49 (range 32–60) in cases and 37 (range 12–52) in controls (t = −3.6019, P = 0.0003). First pass tests of association in EMMAX led to genome-wide deflation of the test statistics (λ = 0.982 SE = 1.74e^−05^) (Fig 1A) that could be directly attributed to the differing age and gender distributions between cases and controls. Modelling the phenotype to adjust for age and gender successfully controlled for this and no genome-wide deviation from the null was subsequently observed (λ = 1.001, SE = 8.7 × 10^−7^) (Fig. 1B). Neither principal components analysis (PCA) (Supplementary Figures 3 & 4), nor tests of proportional identity by state (IBS) variance in PLINK identified significant levels of within or between group genetic variance.

### SNP variants

**Figure 2:**
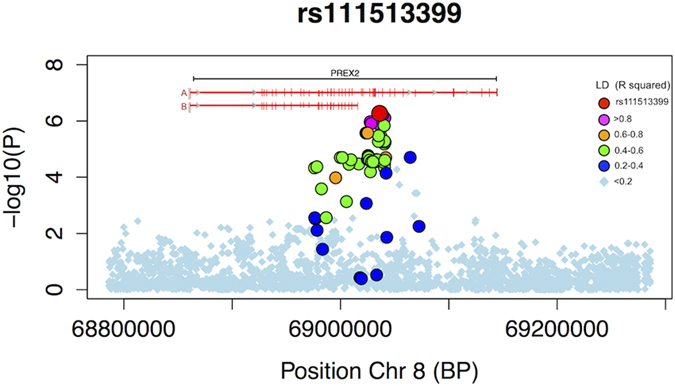
Regional Plot of the most significant index SNP region (rs111513399, *PREX2*). Window size 250 kb. LD with index SNP (R^2^ value) is indicated by colour. LD structure was generated from the GWAS data after imputation. The most significant *PREX2* region coincides with a common splice variation. Known transcript variants (A: NP_079146.2 and B: NP_079446.3) are indicated by horizontal red lines and exons are indicated by crosshatching verticals.

Twenty-seven genomic regions were identified by an index SNP with P_EMMAX_ < 5 × 10^−6^ (Fig. 1B, Supplementary Table 1), although none achieved genome-wide significance (P_EMMAX_ < 5 × 10^−8^). Twelve index SNPs (Table 1 and Supplementary Figure 6) had at least one supporting SNP (either directly genotyped or imputed) in the region that was in high LD (r^2^ > 0.6) with the index. Five of these were located within non-coding regions of genes including *PREX2* (rs111513399), *CTNND* (rs28731189), *PHYH* (rs11258313), *NSUN6* (rs201134023) and *USP6* (rs9895748). The most significant SNP (rs111513399) was in high LD with a number of SNPs with P_EMMAX_ < 1 × 10^−5^ and was located in close proximity to the site of a common splice variation of unknown biological relevance in *PREX2* (Fig. 2).

### Pathways analysis

**Figure 3:**
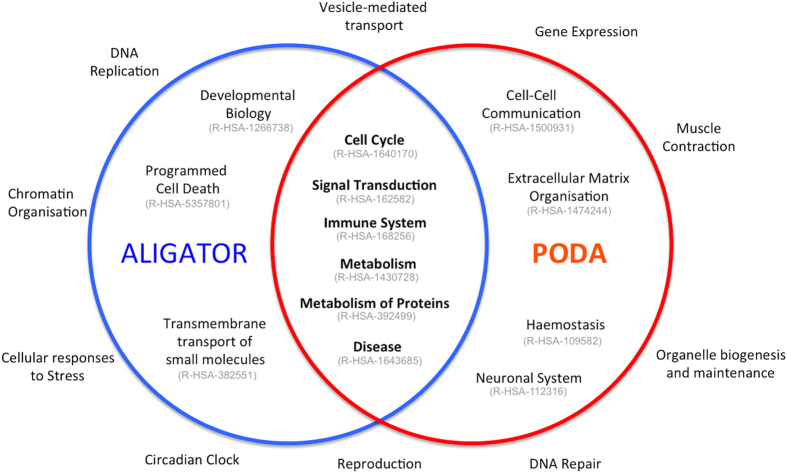
Summary of pathways analysis with ALIGATOR and PODA. Blue circle shows root level Reactome hierarchy event terms and stable identifiers with at least one significant pathway under ALIGATOR. Red circle shows same for PODA analysis. Six branches contained significant pathways under both analyses. Ten branches contained no significant pathways in either analysis.

One-hundred-and-three Reactome pathways had an ALIGATOR P value (P_ALIGATOR_) ≦0.05 after a pre-screening round of 100 permutations. Eighty-four candidate pathways had P after 100,000 permutations (P_100k_) ≦ 0.05 (Table 2). Reactome is arranged in an event hierarchy, a structured relationship table where related pathways share parent terms and where all pathways belong to one of 23 root level event terms. The significant pathways in the ALIGATOR analysis all came under one of nine root level event terms (Cell cycle, Developmental Biology, Disease, Immune system, Metabolism, Metabolism of proteins, Programmed Cell Death, Signal Transduction, Trans-membrane transport of small molecules; Fig. 3). The most significant pathway (P = 0.00001) related to the biology of the innate and adaptive cellular immune response (Table 2), followed by the adaptive response (P = 0.00023) and a number of highly significant and closely related pathways relating to polymorphism in the Fibroblast Growth Factor receptors (FGFR) and their ligands (minimum P = 0.00028). Other significant pathways included mitotic cell cycle processes and several signal transduction pathways, including multiple pathways relating to G-protein coupled receptors (GPCR), Epidermal growth factor receptors (EGFR) and the insulin-like growth factor receptor (IGFR1). The “Disease” pathways appeared to be synonymous with the FGFR pathways.

Fifty-one pathways were significant under PODA with a Pathway Distinction Score P value (DSp) ≦ 0.05 after a pre-screening round of 100 random pathway simulations. Thirty-two pathways had a DSp ≦ 0.05 after 1000 simulations (Table 3). The three most significant pathways in PODA analysis all related to the mitotic cell cycle (DSp = 0.001). The single most significant pathway in PODA was “M phase”, which had a discrimination score of 7.8 and DSp of 0.001. Each unit increase in the S score for a sample was therefore estimated to impart a relative increase in risk of being a TS case of 1.84. Other highly significant pathways related to G protein signalling (DSp = 0.001), events surrounding golgi cisternae pericentriolar stack reorganization during mitosis (DSp = 0.001), GABA receptor activation (DSp = 0.002) and adherens junction organisation (DSp = 0.003). PODA also identified several pathways related to insulin signalling/glucose regulation and the T cell mediated immune response (CD3, ZAP70). All significant PODA pathways came under one of ten root-level Reactome event terms (Cell cycle, Cell-Cell communication, Disease, Extracellular matrix organisation, Haemostasis, Immune System, Metabolism, Metabolism of proteins, Neuronal system, Signalling transduction; Fig. 3).

From a combined list of 111 unique significant pathways, 79 were significant in ALIGATOR only; 27 were significant in PODA only and 5 were significant in both ALIGATOR and PODA. The pathways that were significant in both analyses were “Golgi Cisternae Pericentriolar Stack Reorganization” (P_100k_ = 0.0029, DS_P_ = 0.001), “Mitotic Prophase” (P_100k_ = 0.0059, DS_P_ = 0.002), “Phosphorylation of CD3 and TCR zeta chains” (P_100k_ = 0.049, DS_P_ = 0.005), “Loss of Nlp from mitotic centrosomes” (P_100k_ = 0.0048, DS_P_ = 0.011) and “Loss of proteins required for interphase microtubule organization from the centrosome” (P_100k_ = 0.0029, DS_P_ = 0.011).

There was substantial redundancy and gene overlap between the pathways that were significant in the pathways analysis and hierarchical clustering identified nine clusters of closely related pathways with supporting evidence for TS association in both PODA and ALIGATOR. Each cluster had an approximate UA value > 90 and at least one pathway that was significant in each of PODA and ALIGATOR. GO terms describing the gene content of the 9 clusters are indicated in Table 4.

### Supporting data from independent trachoma Transcriptome Analysis

Pathways level enrichment analysis was performed in four published transcriptome data sets (GSE23705, GSE24383, GSE20436, GSE20430) including two (GSE20436, GSE20430) that included specimens from Gambian individuals who were distinct from those sampled for the GWAS data set. Highly enriched Gene Ontology Biological Processes (GO:BP) and Reactome events were identified in each transcriptome and these are shown in Table 5. The most frequently identified GO:BPs were “Immune Response” and “Cell Cycle”. At pathway level we identified a total of 7 stable Reactome pathways among the 4 GSE mRNA transcriptome series. Overall the analysis of the event hierarchy within Reactome reinforced that the majority of pathways identified were related to either cell cycle (REACT_115566) or the immune system (REACT_6900), where signalling in immune system was the most frequently recognised pathway).

## Discussion

This is the first GWAS study of chlamydial disease. We identified twelve regions of association with P_EMMAX_ < 5 × 10^−6^ for which there was at least one supporting SNP in LD with R^2^ > 0.6. Five of these SNPs were in the regions of genes and some of these may have biological relevance to chlamydial infection and disease (Supplementary Table 2). This study was only modestly powered to detect main effects at the genome-wide level of significance and whilst they are intriguing, the associations that we have observed are at present unconfirmed and will require validation in replication studies; followed by fine mapping in order to identify the underlying causal variants or genes.

**Figure 4:**
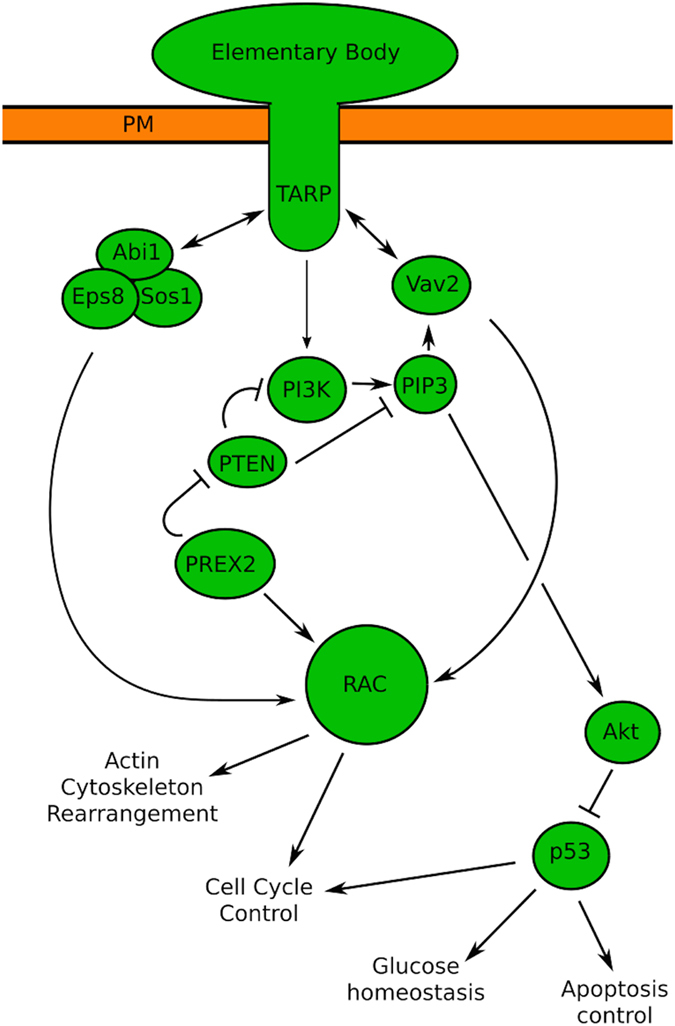
PREX2 is closely involved in processes surrounding TARP mediated Chlamydial entry. Downstream signalling via RAC leads to changes in cell cycle control and actin skeleton rearrangements that facilitate infection. PREX2 can indirectly mediate downstream changes to cell cycle control and glucose homeostasis via RAC and Akt/p53.

The leading SNP-identified candidate gene from this study was *PREX2* (Phosphatidylinositol-3,4,5-trisphosphate-dependent Rac exchange factor 2), a Guanine Nucleotide Exchange Factor (GNEF) and G-protein coupled receptor (G-PCR). PREX2 is known to interact with both Rac and the PI3K inhibitor PTEN^29^ (Fig. 4). Other GNEFs acting upstream of PI3K and Rac have been shown to directly interact with chlamydial TARP^30^, a key molecule that transduces the earliest signals between the chlamydial body and the host cell^31^. PREX2 variants may therefore play a key role in protecting the host cell from Ct entry.

In this study we found no association with rs4149310 (Chr9:107589134) and rs7648467 (Chr3:45936322), two SNPs that were recently predicted to be under selection by trachoma^32^. We note however that the classification of exposed and non-exposed populations in that study better reflects current exposure to ocular infection than historical endemicity for scarring disease. We also did not confirm the findings of a number pre-GWAS era candidate studies^12^,^13^,^15^,^33^,^34^ carried out by our group. No SNP (P_EMMAX_ < 0.01) was detected in any of the genes *IL8, IL10, CSF2, IFNG, HP, CCL8* or *MMP9;* all of which had been previously reported to associate with trachoma. The previous candidate gene studies had small sample sizes as well as a high burden of adjusted testing. They also were unable to correct for cryptic relatedness between participants, which might have inflated the test statistics. Whilst there are many possible reasons for this failure to verify the antecedent studies, we believe that the most probable explanation is that they reported false positive associations. It is however possible that the previous studies reported true positive findings, but that the GWAS SNPs that were genotyped or imputed in the region of these genes were ineffective markers for causative SNPs in the region, as has been demonstrated in another study^22^.

The ALIGATOR/PODA analyses identified a number of highly significantly enriched pathways (Tables 2 & 3) and a joint analysis (Table 4) identified a set of highly enriched GO:BP terms that were prominent among the findings of both ALIGATOR and PODA. The GO:BP terms were compatible with the findings of earlier research in to the biology of chlamydial disease (Supplementary Table 3). The results of these analyses particularly highlighted roles for the immune system, the cell cycle and surface receptor signalling; with metabolism related pathways being a less prominent but still significant feature of the results.

As trachoma is a disease primarily characterised by immune mediated pathology, it is perhaps unsurprising that immune response pathways featured prominently in both the PODA and ALIGATOR analyses. The most significant pathway from ALIGATOR analysis referred to cellular immunity and included both adaptive and innate immune response genes. T cell mediated immunity was also highlighted directly by both methods.

The role of complex immune response genes may still have been underestimated by this study as GWAS has limitations with regards to studying immune response genetics; most particularly because the highly polymorphic gene systems that encode the primary innate and adaptive cellular immunoreceptors, including the Human Leucocyte Antigens (HLA) and Killer-cell Immunoglobulin-like Receptors (KIR) are not well covered by genome-wide SNP arrays. Immune response genes are often inconsistently annotated in ENSEMBL and ENTREZ, with the consequence that they may be poorly represented in the gene lists used in the pathways analysis. The imputation and QC strategies are also likely to reduce the information that is available from complex regions. Previous studies have pointed towards an important, but functionally complex role for both the HLA^11^,^16^,^17^,^19^,^35^ and KIR^11^ systems in TS and to fully appreciate the extent of immunogenetic associations with TS, future studies will be required to perform full sequence resolution genotyping of immunoreceptor genes in large and well powered studies.

G-PCR signalling pathways were significantly enriched in our pathways analyses. In a recent report from a GWAS study of *Chlamydia muridarum* infection in the BXD advanced recombinant inbred mouse^36^, Su and colleagues reported eleven candidate associations with murine oviduct or uterine disease severity. Of these, four were G-PCR signalling molecules^36^. Should the findings from GWAS studies of chlamydial STIs in mice and on-going GWAS in human STI contexts continue to overlap substantially with our own findings, then these are important results providing parallels between tissue tropisms and species.

Many of the significantly enriched pathways (including FGF, hormone receptor and GPCR signalling pathways) converge on events surrounding PI3K and the downstream Akt/mdm2/Caspase9/p53 axis of cell cycle control. A number of papers add support for the p53 tumour suppressor gene being a key player in mediating responses to Ct infection^37^,^38^,^39^,^40^,^41^,^42^,^43^,^44^. This protein may be linked to both G2/M arrest and up-regulation of the pro-fibrotic molecules during Ct infection^45^.

The GWAS and transcriptome data sets of active trachoma (GSE20436, GSE20430) and scarring disease (GSE23705, GSE24383) were obtained using distinct approaches and from separate population samples of trachoma endemic communities in Ethiopia, Tanzania and The Gambia. Both “cell cycle” and “immune system” modules were consistently detected in association with disease in each (Table 5) of the four studies. These findings are complementary to the main findings of the GWAS pathways analysis.

**Figure 5:**
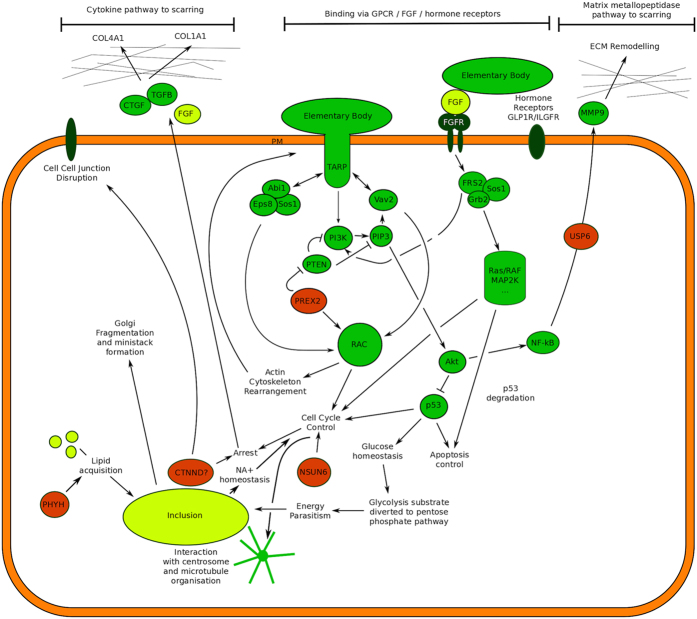
Trachoma associated genes and pathways. Potential roles for candidate genes (red) that were identified through this GWAS are indicated. Various significant cell surface receptors pathways including FGFR, GPCR, ILGFR1 and GLPR1 are linked to cell cycle control by PI3K/Akt/p53 signalling. Chlamydial elementary bodies are known to interact with this system via sos1 and vav2. Downstream signalling from these pathways can lead to actin remodelling (facilitating cell entry), cell cycle arrest and inhibition of apoptosis; all factors that facilitate parasitism. Glucose and sodium ion homeostasis resulting from p53/cell-cycle control may increase nutrient availability to the growing inclusion. Up-regulation of NF_K_B, CTGF, MMP9 and TGFB are potential routes to fibrosis.

Many of the prioritized SNPs, genes and pathways that we identified in this study are known to be functionally linked to one another, as well as to systems that are known to be involved in trachomatous scar formation. A simplified summary of these interactions is presented in Fig. 5, which is derived from our own data and from multiple publicly available open databases (e.g. Genecards, NCBI, Reactome and others). The figure, which shows the convergence of the systems on pathways of cell cycle control, is a model building interpretation of our results that requires experimental validation.

In the context of our data, we propose that innate barriers to the intracellular lifestyle, centring on cell cycle control, may be as important as a well-regulated and proportionate cellular immune response in controlling the pathological sequelae of chlamydial infections.

## Methods

### Ethics statement

Specimens included in this study were obtained from archival stocks of DNA and were used anonymously. All participants had previously consented to the use of their DNA in a study of genetic associations with trachoma (MRC Gambia study codes SCC598, SCC721, SCC729, SCC804, SCC857 and SCC1177) or Chlamydia related tubal infertility (SCC786 and SCC804). Written informed consent was obtained from all adult participants and from a parent/guardian on behalf of those subjects aged under 18 years who wished to take part in the studies. The Ethics Committee of the Gambian Government/Medical Research Council Unit and of the London School of Hygiene & Tropical Medicine approved the antecedent studies for which initial consent was taken. Project approvals for SCC729 and SCC857 were both updated in L 2003.46. All studies were conducted in accordance with the tenets of the Declaration of Helsinki.

### Study population, sampling and ascertainment

The mixed-ethnicity case-control sample was ascertained in multiple rural regions of The Gambia, West Africa. Community screening for trachoma identified cases and each case was asked to identify an unrelated, same-sex member of their community who was also a member of the same ‘kafo’ as the case. A kafo (Mandinka) is a social network of similarly aged individuals of the same gender who are born into the same community.

Samples for DNA analysis were collected from buccal mucosae using sterile cyto-brushes (Part Number F-440151, SLS, Nottingham, UK). DNA extraction was performed using either a salting out procedure or the QIAamp Blood DNA mini kit (Part Number 51106, Qiagen, Manchester, UK). Genomic DNA underwent whole genome amplification by multiple displacement amplification using the Repli-g Midi-Kit (Qiagen, Manchester, UK). Amplified DNA was quantified using PicoGreen (Life Technologies, Paisley, UK), normalised to a standard concentration and analysed by Agilent 2100 Bioanalyzer (Agilent Technologies, Stockport, UK) to verify DNA quality and integrity.

### Trachoma phenotypes

Trachoma was graded in the field using the WHO simplified grading system. The field graders were regularly checked for quality and accuracy of grading as indicated in the manual of operations for the PRET clinical trial^46^. A subject was considered to be a case if they could be defined according to the WHO simplified system as having TS in either eye.

### GWAS genotyping and SNP Quality Control

Specimens (n = 2956) were genotyped at 2,379,855 SNPs using the HumanOmni2.5-8v1_A (Illumina Inc, San Diego, CA. USA). Three genotype-calling algorithms (Illuminus^47^, GenCall^48^ and GenoSNP^49^) were used on the initial set of SNPs (n = 2,379,855). Data from each algorithm was filtered to retain only SNPs with a call rate ≥ 0.98. The number of SNPs retained was 1,403,253 in Illuminus, 219,259 in GenCall and 929,088 in GenoSNP (Supplementary Figure 1). To obtain a merged set of SNPs, all genotypes that matched across call-sets were retained, whilst those that mismatched between call-sets were set to missing (Supplementary Figure 2). Genotypes that were present in one call set and missing in others were also retained (Supplementary Figure 2). The merged SNP set contained 1,467,876 SNPs. Merged SNPs were finally retained for analysis if (a) the call rate ≥ 0.99 and (b) the Hardy-Weinberg equilibrium P value < 5 × 10^−8^. 1,457,295 directly genotyped SNPs were retained after quality control (QC) (Supplementary Figure 1).

### Specimen Quality Control and statistical power

Individuals were removed if they were identified as being outliers because they had: (a) > 5% missing genotype data. (b) Genome-wide heterozygosity > 1.96 × standard deviation of the sample wide average genome-wide heterozygosity. (c) Average identity by state with all other individuals > 0.05. (d) Identity by descent sharing with another individual of two alleles at all loci. (e) Identity by state with the fifth nearest neighbour with Z < −4 compared to the mean IBS of all possible pairs. (f) Unresolved gender mismatch between sex chromosome genotypes and clinical record. After these QC steps, 2621 specimens were retained (Supplementary Figure 1).

Tests of proportional IBS variance were performed in PLINK. Analysis of population stratification by supervised PCA was implemented in Eigenstrat smartPCA^50^,^51^ (Supplementary Methods and Supplementary Figures 3 & 4). Familial relationships within the sample were identified using an analysis of pairwise identity by state/allele sharing (Supplementary Figure 5) in PLINK and R.

The STATA “power twoproportions” command was used to estimate the power of the study to detect genome-wide significant (α < 1 × 10^−8^) associations. At this level of significance, a study of 1090 cases and 1531 controls has 80% power to detect allele frequency odds ratios (OR) of 2.61, 2.11, 1.81 and 1.72 for minor allele frequencies (MAF) in the control group of 0.05, 0.1, 0.2 and 0.3 respectively.

### Imputation

Imputation was carried out as described by Howie *et al.*^52^. Shapeit^53^,^54^ was used to pre-phase using data from HapMap Phase II, build 37. Imputation was performed with IMPUTE2 52,55 and utilized data from 1092 reference samples included in the worldwide 1000 Genomes phase I data set^56^. Post imputation filtering was based on Southam *et al.*^57^. SNPs with an IMPUTE2 info score <0.8 and/or MAF <0.01 were discarded. In total, 28,755,674 SNPs were imputed. After filtering for quality 11,851,747 SNPs were retained (Supplementary Figure 1).

### Tests of association

Tests of association of SNPs with age and sex corrected trachoma phenotypes were performed using EMMAX^58^ and were informed by an IBS ‘kinship’ matrix (*.hIBS.kinf format) generated from the directly genotyped SNP data in PLINK. In order to compensate for the relatively lower age distribution of the control set, we modeled the phenotype with age and gender under logistic regression and then used the residuals of this analysis as an age and gender corrected TS phenotype in the EMMAX test. Highlighted SNPs were annotated using SNPNexus (http://snp-nexus.org).

### Pathways analyses

ALIGATOR^26^ was performed using the SNPath R package (linchen.fhcrc.org/grass.html) and 1345 Reactome pathways^59^ (www.reactome.org, accessed April 2013), also known as “events”. The input data were the P values from EMMAX tests of association. These were thinned to remove SNPs that were >20 kb away from any gene in a list of ~17,000 genes that were consistently cross-referenced between Entrez and Ensembl and for which a HUGO gene name had been assigned (http://www.gettinggeneticsdone.com/2011_06_01_archive.html). Genes in this list are likely to be included in pathway lists (such as Reactome), whilst genes with inconsistent cross-referencing are unlikely to be included. ALIGATOR counts the number of genes in a pathway that contain a SNP with an EMMAX P value (P_EMMAX_) value more extreme than a nominally significant threshold value. We set this threshold at P_EMMAX_ < 0.001 (Supplementary Methods and Supplementary Table 4). Pathways of Distinction Analysis (PODA)^25^ was performed (Supplementary Methods) on directly genotyped GWAS data using 1345 Reactome^59^ pathways and the PODA script for R (http://braun.tx0.org/PODA/).

A proportional gene content intersection between the members of a combined list of significant pathways from ALIGATOR and PODA was used to detect functional redundancy and gene content overlap in significant pathways. For each possible pair of pathways, the number of intersecting genes between pathways was divided by the number of genes in the union of the two pathways. This generated a distance matrix that was subjected to hierarchical clustering with multi-scale bootstrap sampling using the pvclust R package (Suzuki & Shimodaira 2014, http://CRAN.R-project.org/package = pvclust). Clusters of interest were identified as having an approximate unbiased alpha (UA) value greater than 90 and containing at least one pathway that was significant in each of the PODA and ALIGATOR results. UA is a probability measure where UA = 90 indicates that there is 90% confidence that the pathways form a cluster. The combined gene content from all pathways in each cluster were then functionally annotated with Gene Ontology: Biological Process terms using DAVID Bioinformatics Resources 6.7 (accessed 05/2015)^60^.

#### Supporting data from independent trachoma Transcriptome Analysis

We sought to support the GWAS pathway level analysis features by performing a pathways level analysis on GEO deposited trachoma data-sets from the Gambia, Tanzania and Ethiopia (Table 5). Each transcriptome data set was reanalysed in a standardised way in which differential expression was calculated using GEO2R (Limma)^61^. Pearson correlation network graphs were then generated and partitioned into co-expression clusters by Markov-chain clustering using Biolayout express 3D^62^. The top level features of complete networks and co-expression clusters were then extracted by interrogation using NCBI DAVID v6.7 and where 5% FDR significant pathways were identified in Reactome via DAVID, these gene sets were directly queried within the Reactome database (accessed April 2013) to describe pathway hierarchical structure and fine level features from the same pathway database used in the GWAS analysis. For each data set we obtained an FDR adjusted p-value queried against the 1345 Reactome pathways. For each of the top 12 Reactome pathways identified by ALIGATOR/PoDA (Tables 2 and 3) we identified the event hierarchy and the associated p-value of the sub-pathway from transcriptome gene-set enrichment analysis.

### Data Dissemination

Managed access to the individual-level genotypes, TS phenotypes, age and gender data will available to all appropriately qualified researchers from academia, charitable organizations and private companies in the UK or abroad under the terms of the Wellcome Trust Community Access Policy and via the European Genome-phenome Archive (EGA at EMBL-EBI). Details of how to access the data will be available on the project information page at the EGA website. The EGA study accession number for this project is EGAS00001001516.

## Additional Information

**Accession codes:** The EGA study accession number for this project is EGAS00001001516.

**How to cite this article**: Roberts, C. *et al.* Conjunctival fibrosis and the innate barriers to Chlamydia trachomatis intracellular infection: a genome wide association study. *Sci. Rep.* **5**, 17447; doi: 10.1038/srep17447 (2015).

## Supplementary Material

Supplementary Information

## Figures and Tables

**Table 1:** Index SNPs (P_emmax_ ≤ 5 × 10^−6^, at least one supporting SNP in LD with R^2^ > 0.6) for candidate associated regions.

RSID	CHR	POSITION Assembly 3GRCh37.p13	ALLELES (effect allele bold)	Freq EA	Type	P	OR*,**	Within gene
rs116828186	3	18854524	A,**G**	0.027	Genotyped	1.37E-06	0.824	—
rs28731189	5	11212170	C,**T**	0.011	Imputed	1.42E-06	0.736	CTNND2
rs74291850	5	29577370	T,**C**	0.022	Imputed	4.63E-07	1.291	—
rs187984259	5	73681906	T,**C**	0.016	Imputed	1.43E-06	0.758	—
rs80317841	6	132398394	**G**,C	0.84	Genotyped	1.97E-06	1.092	—
rs116141145	7	97249265	A,**G**	0.037	Imputed	1.24E-06	1.190	—
rs111513399	8	69036056	A,**G**	0.113	Imputed	5.38E-07	1.114	PREX2
rs11258313	10	13339766	G,**A**	0.233	Imputed	1.35E-06	1.101	PHYH
rs12774519	10	18925493	C,**G**	0.191	Imputed	2.06E-06	1.086	NSUN6
rs9895748	17	5053598	T,**A**	0.068	Imputed	1.72E-06	0.872	USP6
rs62079945	18	10247566	A,**C**	0.048	Imputed	1.22E-06	0.842	—
rs6033064	20	1175527	**T**,C	0.803	Imputed	9.94E-07	0.909	—

^*^Expected frequency of the effect allele.

^**^The OR indicates the estimated allele frequency odds ratio for the effect allele. Values less than one indicate that the effect allele is less common in cases than controls and vice versa.

**Table 2:** Reactome pathways with significant enrichment in scarring trachoma: ALIGATOR analysis.

Pathway Name	P_100k_
Immunoregulatory interactions between a Lymphoid and a non Lymphoid cell	0.00001
Adaptive Immune System	0.00023
FGFR3b ligand binding and activation	0.00028
FGFR2c ligand binding and activation	0.00029
FGFR4 ligand binding and activation	0.00048
FGFR3 ligand binding and activation	0.00050
FGFR3c ligand binding and activation	0.00050
Signaling by activated point mutants of FGFR3	0.00050
Signaling by FGFR3 mutants	0.00050
FGFR ligand binding and activation	0.00078
Activated point mutants of FGFR2	0.00117
FGFR2 ligand binding and activation	0.00117
Signaling by FGFR2 mutants	0.00117
Phospholipase C mediated cascade	0.00139
Glucagon type ligand receptors	0.00204
Class B2 Secretin family receptors	0.00212
Mitotic G2 G2M phases	0.00262
Immune System	0.00270
Golgi Cisternae Pericentriolar Stack Reorganization	0.00294
Androgen biosynthesis	0.00376
activated TAK1 mediates p38 MAPK activation	0.00408
SHC mediated cascade	0.00450
Loss of Nlp from mitotic centrosomes	0.00484
Loss of proteins required for interphase microtubule organization from the centrosome	0.00484
G protein betagamma signalling	0.00488
G2M Transition	0.00515
Downstream signal transduction	0.00546
Alpha defensins	0.00576
Mitotic Prophase	0.00586
G betagamma signalling through PI3Kgamma	0.00599
Signaling by PDGF	0.00642
Polo like kinase mediated events	0.00649
Activation of NOXA and translocation to mitochondria	0.00747
FGFR1c ligand binding and activation	0.00967
Signaling by activated point mutants of FGFR1	0.00967
PD 1 signaling	0.01000
Cell Cycle Mitotic	0.01036
Centrosome maturation	0.01103
Recruitment of mitotic centrosome proteins and complexes	0.01103
Negative regulation of FGFR signaling	0.01125
FRS2 mediated cascade	0.01159
Opioid Signalling	0.01291
DAP12 interactions	0.01315
Insulin receptor signalling cascade	0.01502
Effects of PIP2 hydrolysis	0.01510
Constitutive PI3KAKT Signaling in Cancer	0.01641
FGFR1 ligand binding and activation	0.01674
Intrinsic Pathway for Apoptosis	0.01743
DAP12 signaling	0.01814
IRS related events	0.01867
Amino acid and oligopeptide SLC transporters	0.01903
G alpha q signalling events	0.01928
Transport of inorganic cationsanions and amino acidsoligopeptides	0.02043
Translocation of ZAP 70 to Immunological synapse	0.02068
Amino acid transport across the plasma membrane	0.02111
Signaling by Insulin receptor	0.02145
Costimulation by the CD28 family	0.02279
IRS related events triggered by IGF1R	0.02305
Downstream signaling of activated FGFR	0.02430
Chondroitin sulfatedermatan sulfate metabolism	0.02463
PI3K Cascade	0.02494
TAK1 activates NFkB by phosphorylation and activation of IKKs complex	0.02825
IGF1R signaling cascade	0.02859
Signaling by Type 1 Insulin like Growth Factor 1 Receptor IGF1R	0.02859
Leukotriene receptors	0.02905
Cell Cycle	0.02944
Activation of BH3 only proteins	0.03137
Synthesis Secretion and Inactivation of Glucagon like Peptide 1 GLP 1	0.03145
Cyclin B2 mediated events	0.03186
IRS mediated signalling	0.03214
CTLA4 inhibitory signaling	0.03366
Acyl chain remodeling of DAG and TAG	0.03374
Signaling by EGFR	0.03471
A tetrasaccharide linker sequence is required for GAG synthesis	0.03491
Signaling by NODAL	0.03554
Signaling by EGFR in Cancer	0.03722
Gastrin CREB signalling pathway via PKC and MAPK	0.03909
HCN channels	0.04039
GAB1 signalosome	0.04156
Electric Transmission Across Gap Junctions	0.04363
Transmission across Electrical Synapses	0.04363
Signaling by ERBB2	0.04826
Signaling by Activin	0.04877
Phosphorylation of CD3 and TCR zeta chains	0.04903

**Table 3:** Reactome pathways with significant enrichment in scarring trachoma: PODA analysis.

Name	DS	DSp	OR
M Phase	7.774	0.001	1.842
Mitotic G2-G2/M phases	6.935	0.001	1.649
G2/M Transition	6.663	0.001	1.623
G alpha (z) signalling events	4.803	0.001	1.525
Golgi Cisternae Pericentriolar Stack Reorganization	4.146	0.001	1.239
Proteolytic cleavage of SNARE complex proteins	3.955	0.001	1.230
GABA receptor activation	5.616	0.002	1.595
Mitotic Prophase	5.246	0.002	1.392
Adherens junctions interactions	4.990	0.003	1.547
Cell-cell junction organization	5.100	0.004	1.641
Synthesis of very long-chain fatty acyl-CoAs	3.833	0.005	1.323
Phosphorylation of CD3 and TCR zeta chains	3.668	0.005	1.268
G-protein beta:gamma signalling	4.159	0.006	1.388
Calmodulin induced events	4.020	0.007	1.441
Glucagon signaling in metabolic regulation	4.266	0.007	1.425
Loss of Nlp from mitotic centrosomes	5.181	0.011	1.551
Loss of proteins required for interphase microtubule organization from the centrosome	5.181	0.011	1.551
G beta:gamma signalling through PI3Kgamma	3.948	0.011	1.350
Botulinum neurotoxicity	3.608	0.012	1.238
Inhibition of Insulin Secretion by Adrenaline/Noradrenaline	3.689	0.013	1.332
Synthesis of PIPs at the late endosome membrane	2.798	0.014	1.169
Insulin Processing	3.834	0.015	1.346
Mitotic Metaphase and Anaphase	7.029	0.018	1.760
PLC beta mediated events	4.205	0.024	1.541
Mitotic M-M/G1 phases	7.461	0.027	1.903
PKA activation in glucagon signalling	3.243	0.031	1.322
GPVI-mediated activation cascade	3.887	0.035	1.403
Translocation of ZAP-70 to Immunological synapse	2.631	0.035	1.177
Assembly of collagen fibrils and other multimeric structures	4.403	0.046	1.454
Inhibition of the proteolytic activity of APC/C required for the onset of anaphase by mitotic spindle checkpoint components	3.024	0.048	1.261
Adenylate cyclase inhibitory pathway	2.637	0.048	1.247
Inhibition of adenylate cyclase_pathway	2.637	0.048	1.247

DS: Discrimination Score. DSp: P value for DS based on 1000 permutations, OR: Odds ratio of being a case, per unit increase in S score.

**Table 4:** Gene Ontology terms associated with pathways of significance in PODA and ALIGATOR.

GO Terms: Biological Process	#genes in list	P value for GO term*	UA value for cluster**
Microtubule-based Process	443	2.3 × 10^−21^	100
G-protein coupled Receptor protein signalling pathway	353	1.3 × 10^−116^	91
Cellular response to hormone stimulus	53	2.5 × 10^−24^	100
Cell surface receptor linked signal transduction	147	2.4 × 10^−24^	97
Cell Cycle	1380	4.8 × 10^−189^	100
Regulation of T cell activation	82	2.1 × 10^−15^	100
Sodium Ion Transport	175	2.1 × 10^−61^	100
Phosphorous metabolic process	36	4.3 × 10^−2^	98
Regulation of phosphorylation	131	2.6 × 10^−13^	97

^*^Modified Fisher exact P, EASE SCORE.

^**^Unbiased Alpha.

**Table 5:** Matrix of Gene Ontology: Biological Process for trachoma transcriptome co-expression modules (FDR P values).

Transcriptome series				
GEO Accession Number	GSE23705	GSE24383	GSE20436	GSE20430
Phenotype	Trichiasis	Scarring	Active trachoma	Active trachoma
Country of origin	Ethiopia	Tanzania	Gambia	Gambia
Number of Specimens in Series	42	42	60	29
Specimen Type	Conjunctival swab			
Disease States (see footer)	a	b	c	d
Platform	Illumina WG-6 v3.0	Illumina WG-6 v3.0	Affymetrix U133A plus 2.0	Affymetrix HG-focused
Pathway Enrichment (FDR P values)				
Cell cycle	1.4 × 10^−32^	—	1.7 × 10^−64^	—
Cell cycle - M Phase	—	—	—	1.9 × 10^−19^
Regulation of Apoptosis	—	1.9 × 10^−9^	—	—
Immune Response	5.6 × 10^−4^	3.1 × 10^−7^	3.4 × 10^−17^	2.3 × 10^−3^
Lymphocyte activation	—	—	4 × 10^−26^	9.7 × 10^−10^
Cell activation	—	—	—	8.9 × 10^−5^
Defense Response	—	1 × 10^−10^	—	6 × 10^−6^
Response to virus	—	9.3 × 10^−26^	2.5 × 10^−11^	—
T cell activation	—	2 × 10^−12^	—	—
Chemotaxis	—	—	—	3 × 10^−5^
Ectoderm development	—	2.1 × 10^−12^	4.9 × 10^−8^	—
Transcription	—	—	1.7 × 10^−8^	—
Translation	2.1 × 10^−21^	—	—	—
Translational elongation	4 × 10^−14^	8.7 × 10^−34^	—	—

^a^Trachomatous Trichiasis with and without inflammation (no current C. trachomatis infection).

^b^Trachomatous conjunctival scarring with and without inflammation (no current C. trachomatis infection).

^c^Active follicular disease with and without C. trachomatis infection.

^d^Active follicular disease with and without C. trachomatis infection.
