# Supplementary material for: Conjunctival fibrosis and the innate barriers to Chlamydia trachomatis intracellular infection: a genome wide association study
Source: Sci Rep. 2015 Nov 30;5:17447. doi: 10.1038/srep17447 (PMC4663496; doi:10.1038/srep17447)
Supplement: Supplementary Information [file srep17447-s1.pdf]

Supplementary Materials

Conjunctival fibrosis and the innate barriers to Chlamydia trachomatis intracellular infection: a genome wide association study

Chrissy h. Roberts[1#*], Christopher S Franklin[2#], Pateh Makalo[3], Hassan Joof[3], Isatou Sarr[3], Olaimatu S Mahdi[3], Ansumana Sillah[4], Momodou Bah[5], Felicity Payne[2] Anna E Jeffreys[7], William Bottomley[2], Angels Natividad[1], Sandra Molina-Gonzalez[1], Sarah E Burr[1,3], Mark Preston[1], Dominic Kwiatkowski[27], Kirk A Rockett[7], Taane G Clark[1], Matthew J Burton[6], David CW Mabey[1], Robin Bailey[1], Inês Barroso[2,8,9&] and Martin J Holland[1&]

1. London School of Hygiene and Tropical Medicine, London, UK
2. Wellcome Trust Sanger Institute, Hinxton, UK
3. Medical Research Council Unit, The Gambia, Atlantic Boulevard, Fajara, The Gambia
4. National Eye Care Programme, Gambian Ministry of Health, Banjul, The Gambia
5. Sightsavers International, The Gambia. Kairaba Avenue, Banjul, The Gambia
6. International Centre for Eye Health, London, UK
7. Wellcome Trust Centre for Human Genetics, Oxford, UK
8. University of Cambridge Metabolic Research Laboratories, Wellcome Trust-MRC Institute of Metabolic Science, Cambridge, UK
9. NIHR Cambridge Biomedical Research Centre, Cambridge, UK

# and & : These authors contributed equally to the work

* Corresponding author.
Mailing address :
Clinical Research Department, London School of Hygiene and Tropical Medicine, Keppel St. London, UK WC1E 7HT
Phone : +44 (0) 20 7927 2913
E-mail : chrissy.roberts@lshtm.ac.uk

Supplementary figure 1 : Quality control, base-calling, SNP and sample filtering and imputation.

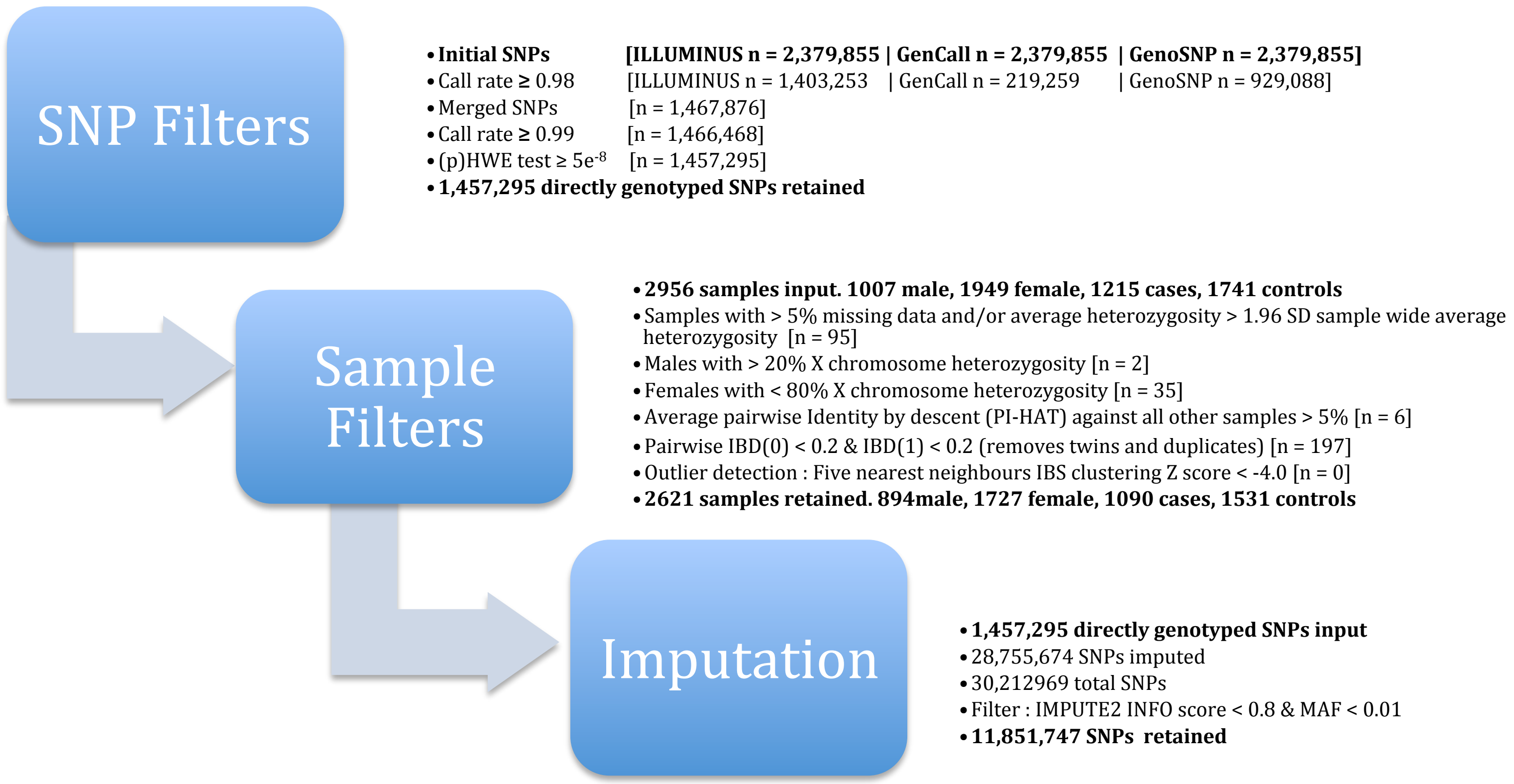

Supplementary figure 2: Intersection of high call rate SNPs between three genotype-calling algorithms.

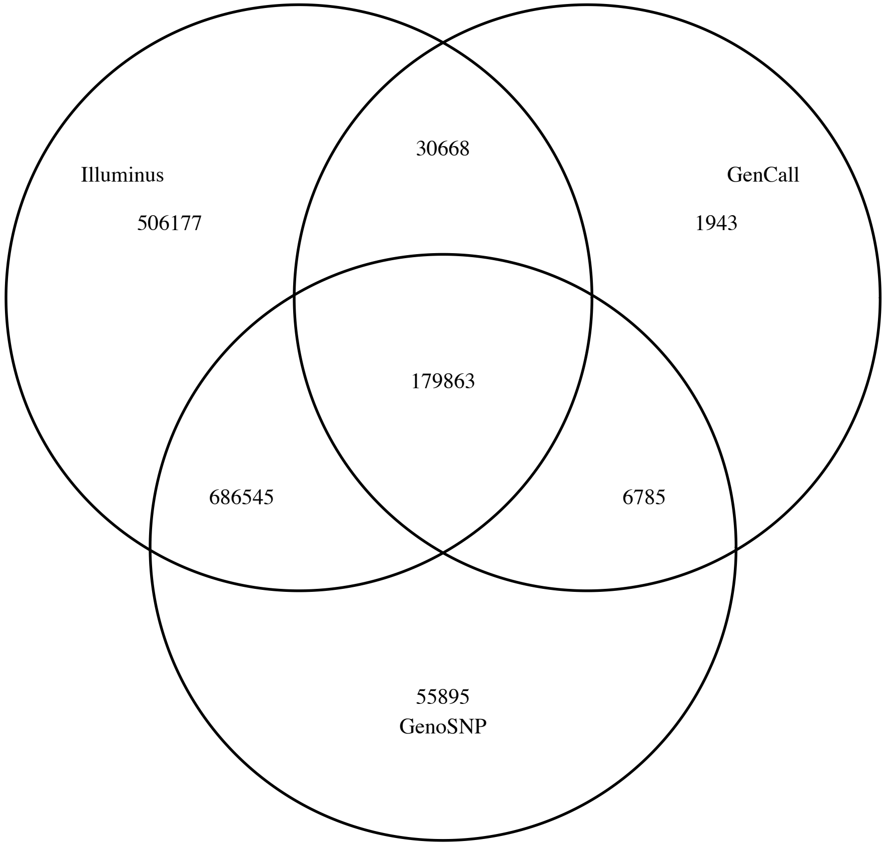

Supplementary Figure 3. Principle Components Analysis using 1000G super-population references. (A) Principle components 1&2 and (B) Principle components 3&4.
Population codes: AFR : African, AMR : Ad mixed American, ASN : East Asian, EUR : European and SAN : South Asian.

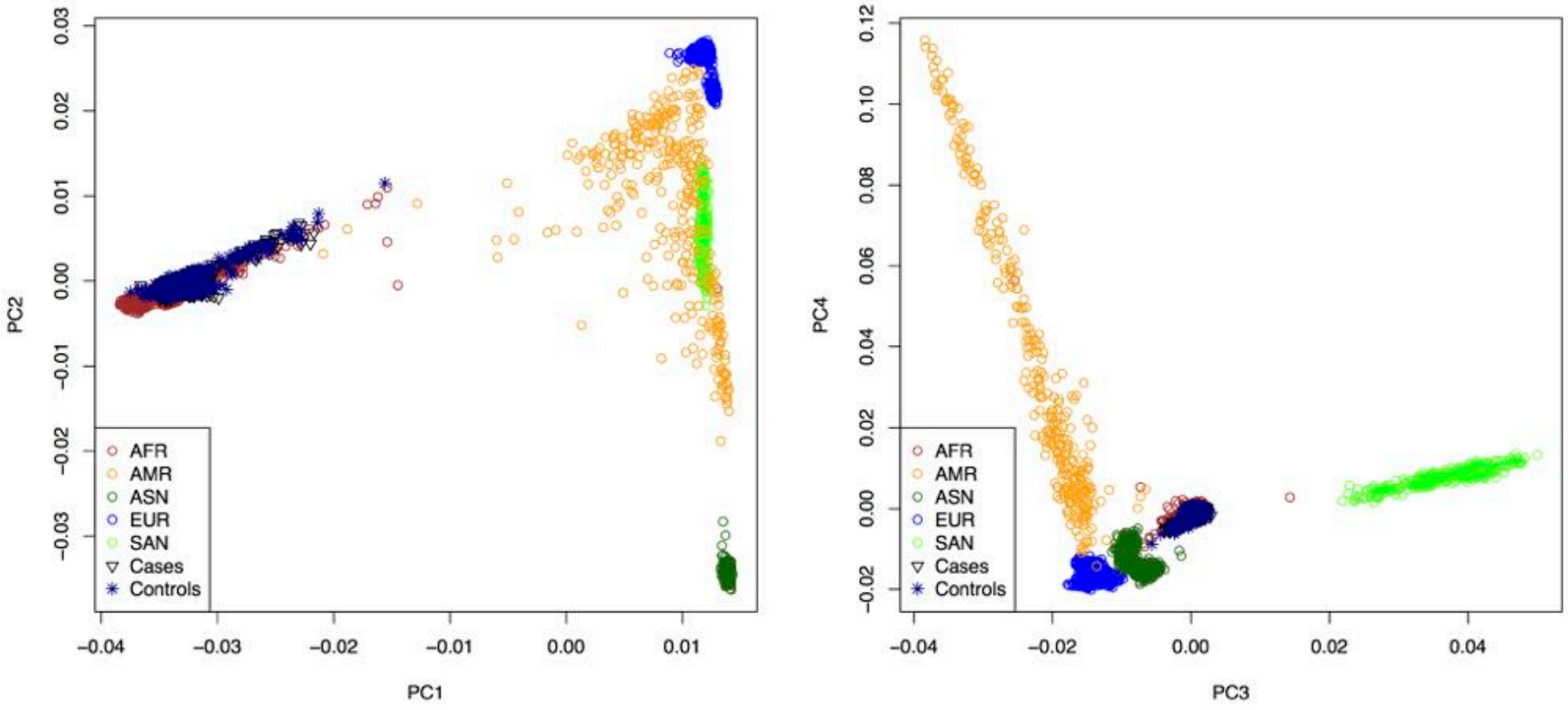

Supplementary Figure 4. Principle Components Analysis using African reference populations. (A) Principle components 1&2 and (B) Principle components 3&4. ACB : African Carribean, ASW : African American in Southwest USA, ESN : Esan in Nigeria, GWD : Gambian in Western Division, LWK : Luhya in Webuye, Kenya, MSL : Mende in Sierra Leone and YRI : Yoruba in Ibadan, Nigeria.

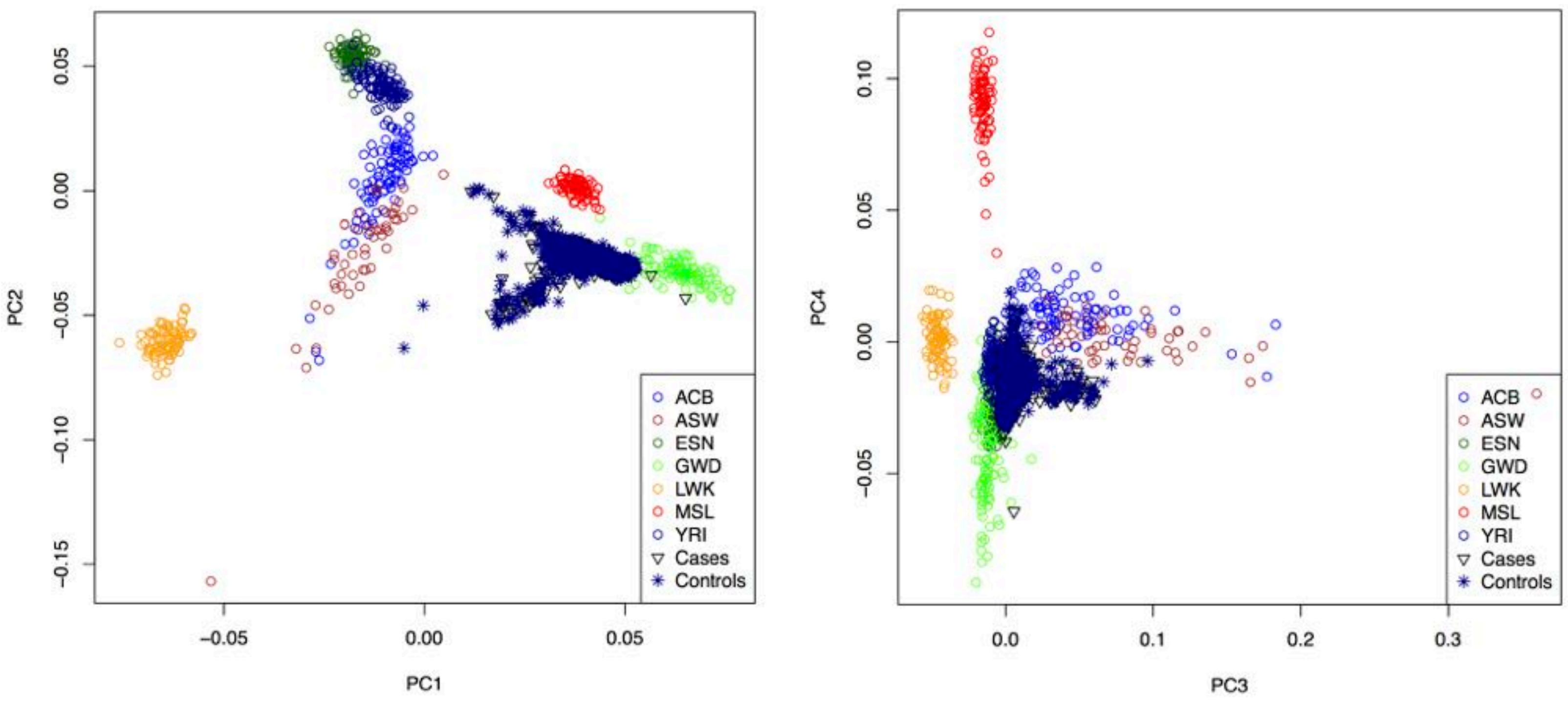

Supplementary figure 5 : Pairwise estimates of proportion of alleles shared with IBD.
Red : Duplicates and Twins, Yellow : Parent-Offspring pairs, Green : Full-siblings, Pink : Second degree relatives, Light blue : Third/fourth degree relatives, Purple : More distant relationships. Approximately $2.5e^6$ pairs with IBD(0 alleles) > 0.9 are not shown.

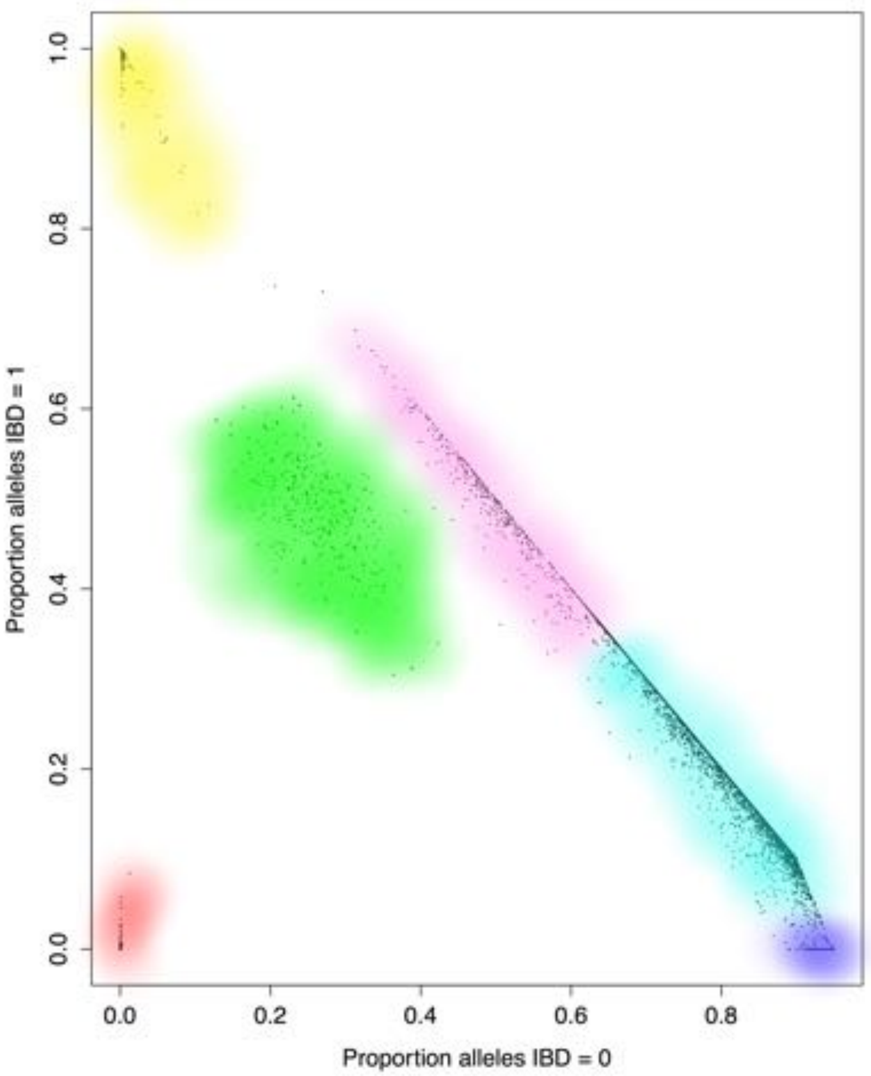

Supplementary figure 6 : Regional Plots of 12 SNPs with $P_{EMMAX} < 1 \times 10^{-6}$ and at least one SNP in region with $R^2 > 0.6$. Window size 250 kb. Dark red point is the SNP of interest. Pairwise LD of index SNP to other SNPs in region is indicated by coloration of points. LD structure was generated from imputed Gambian trachoma data.

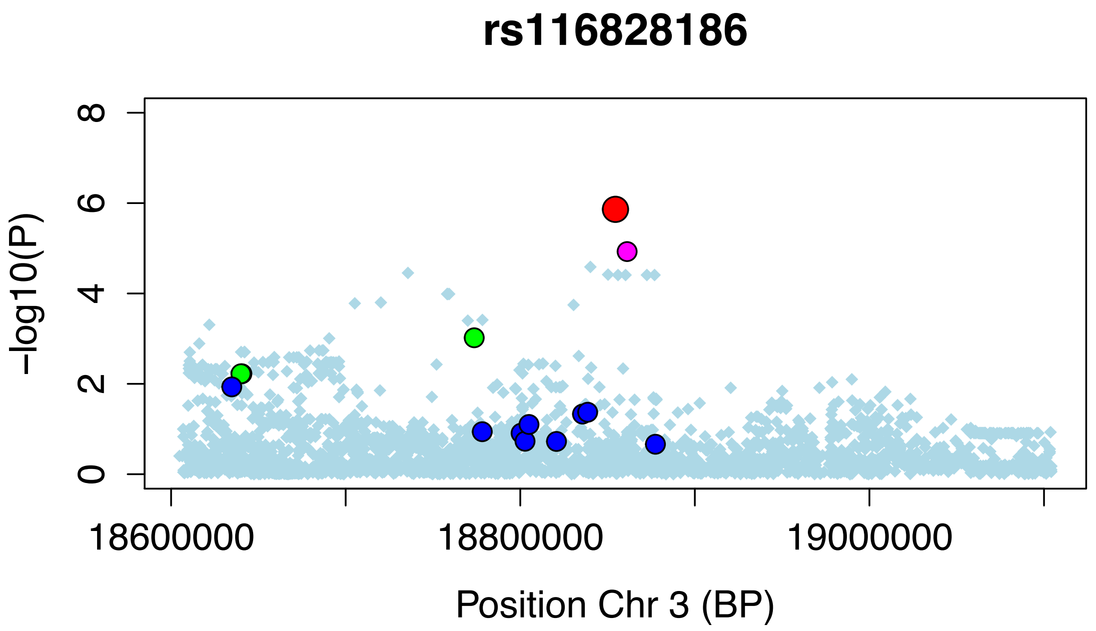

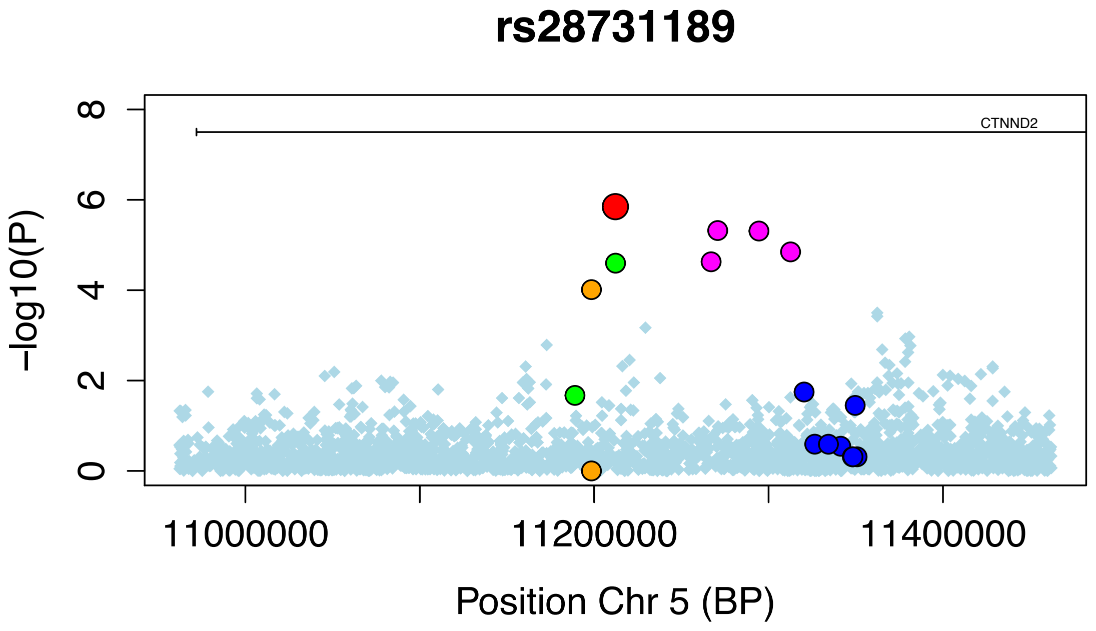

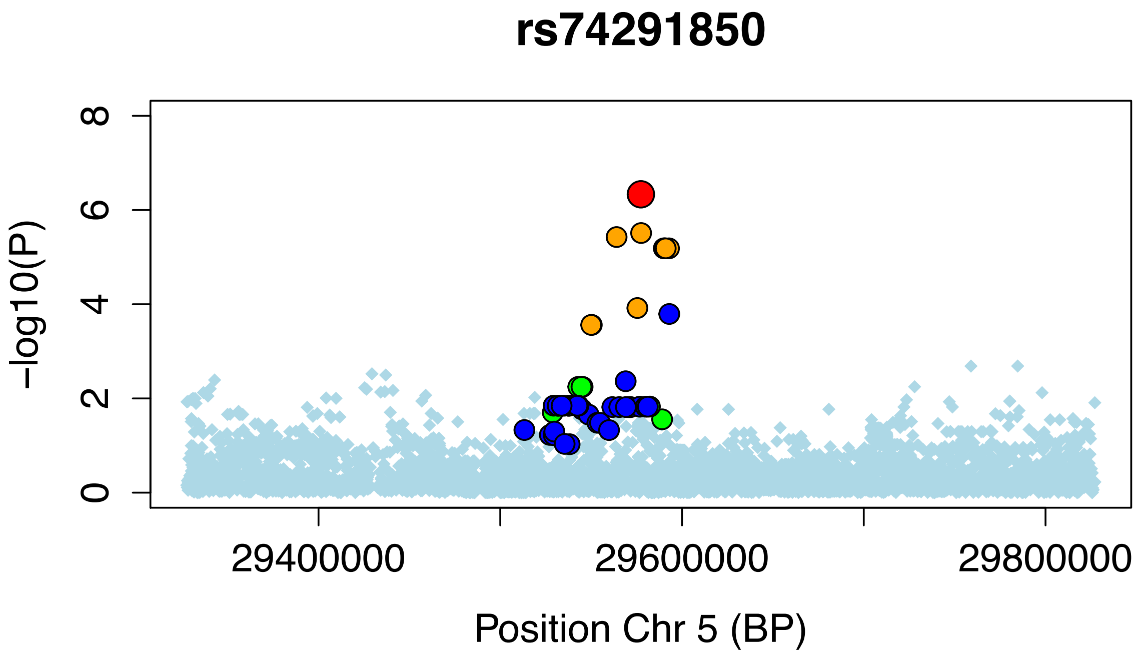
rs74291850
−log10(P)
0
2
4
6
8
29400000
29600000
29800000
Position Chr 5 (BP)

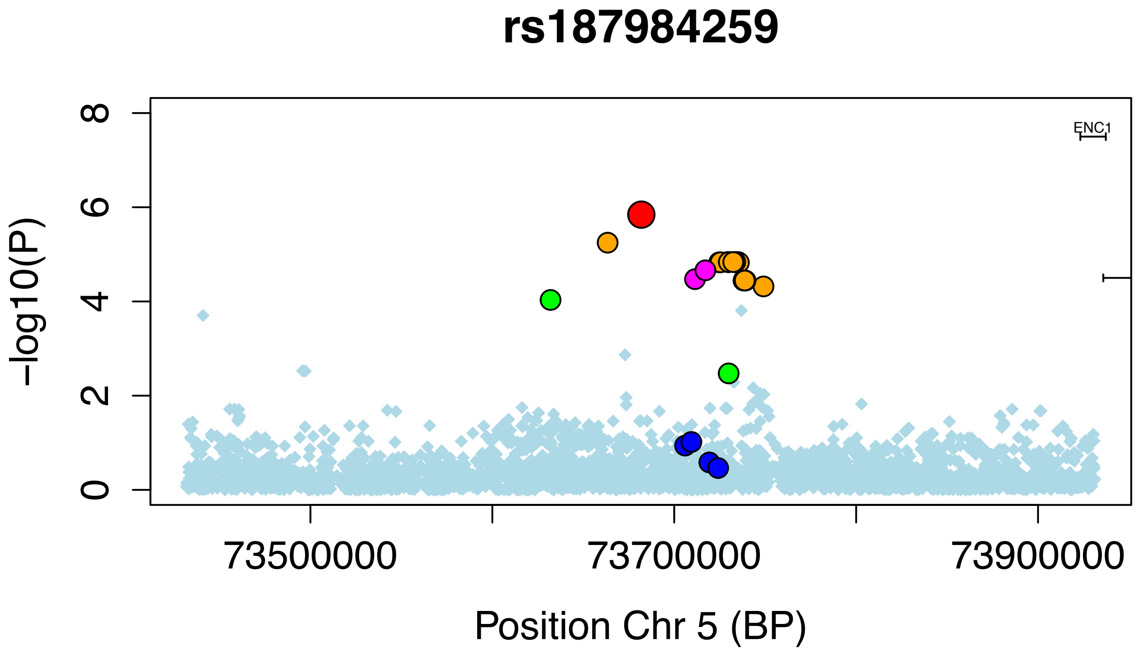
rs187984259
−log10(P)
0
2
4
6
8
ENC1
73500000
73700000
73900000
Position Chr 5 (BP)

rs80317841

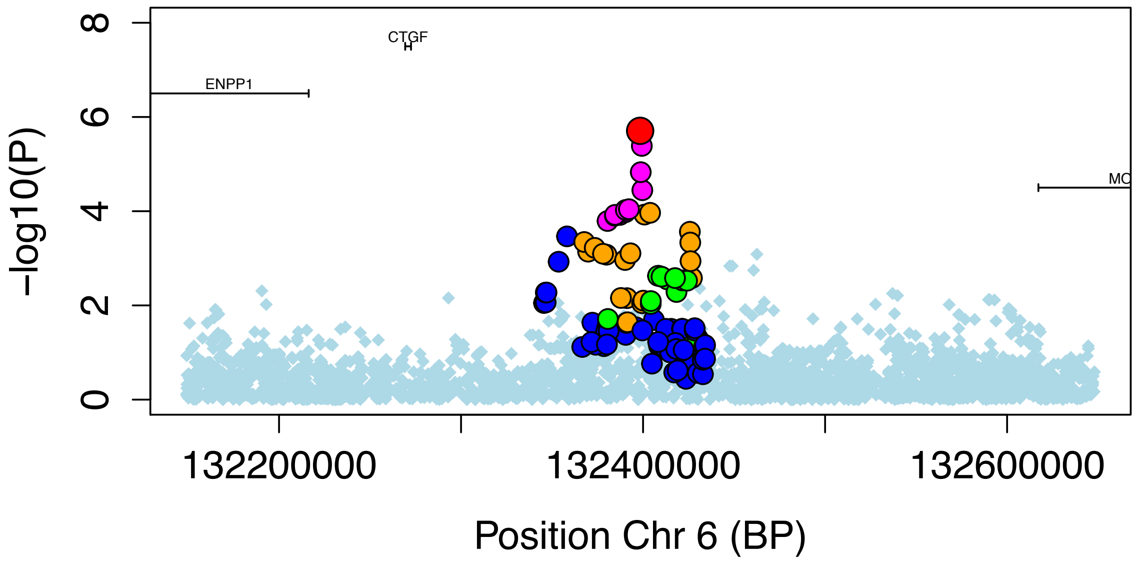

rs116141145

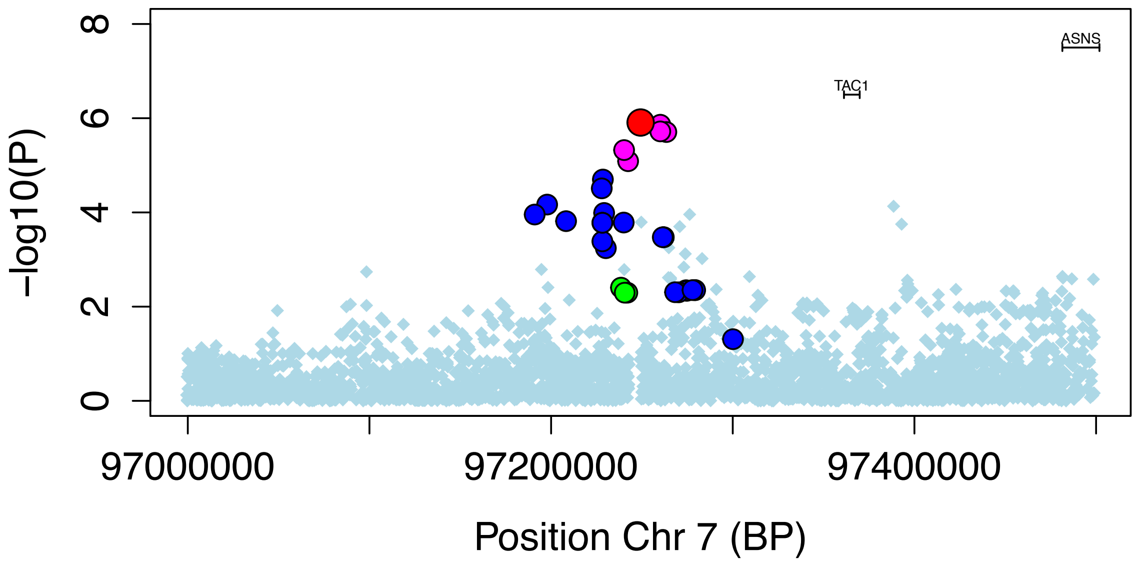

rs111513399

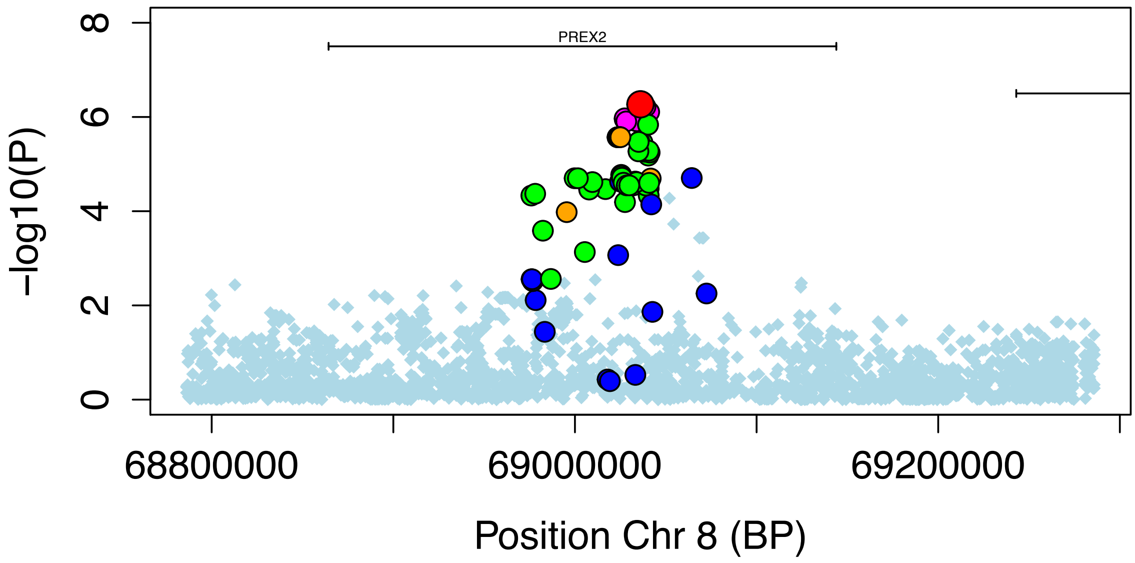

rs11258313

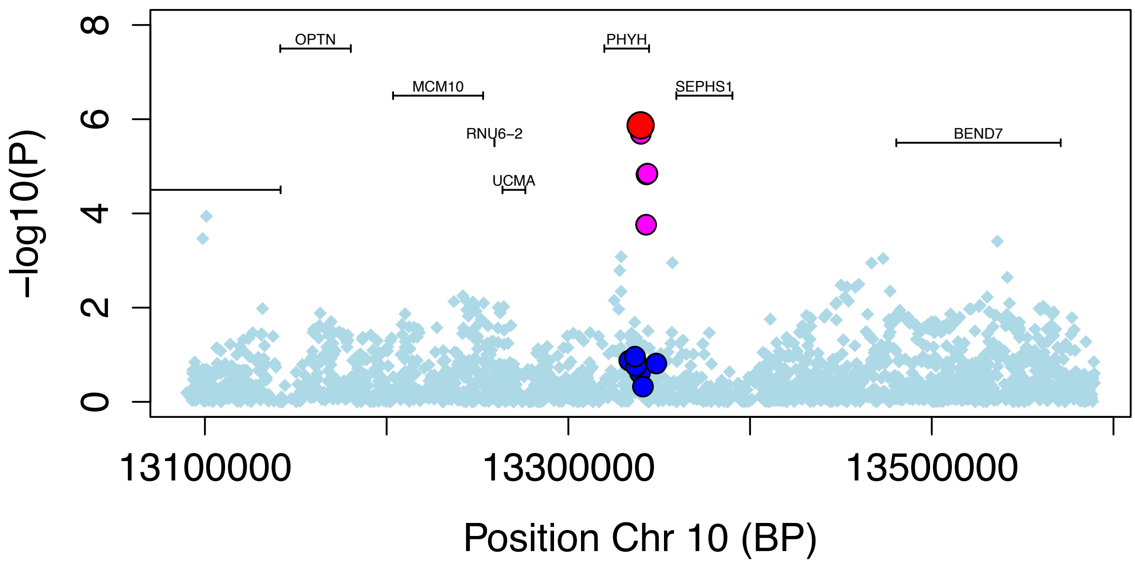

rs12774519

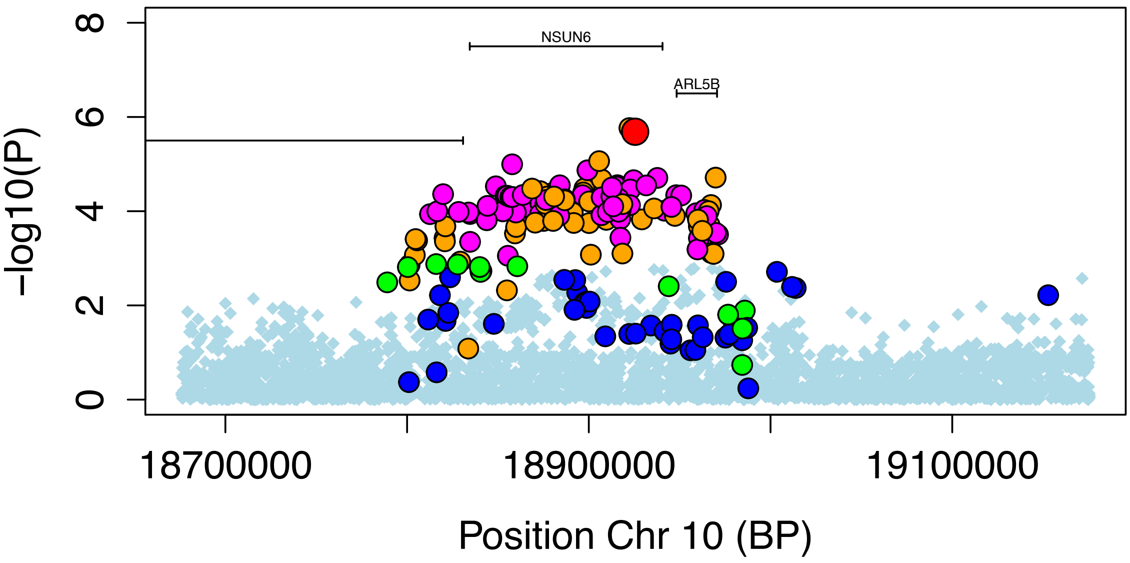

rs9895748

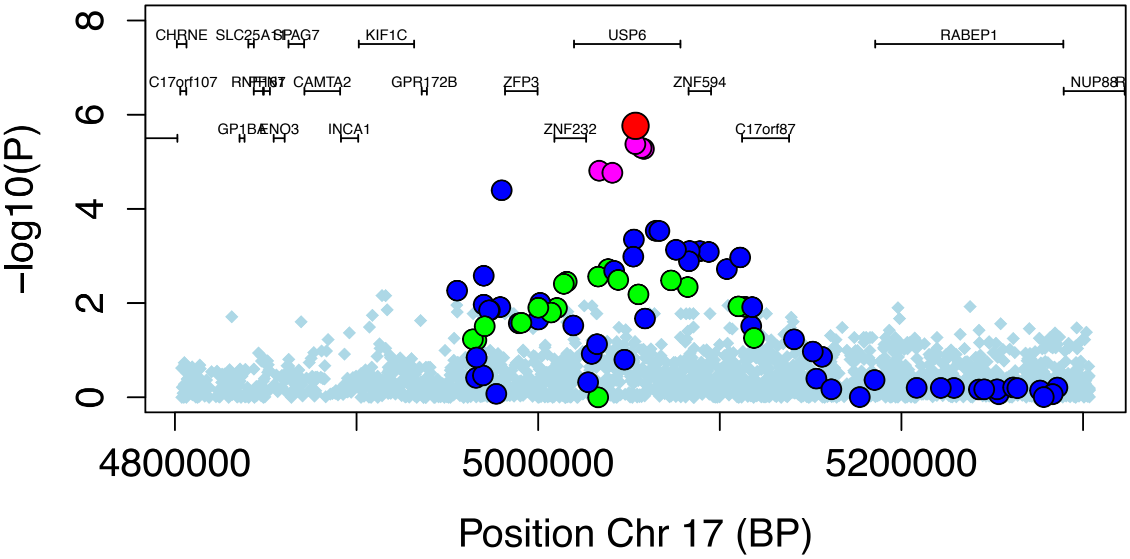

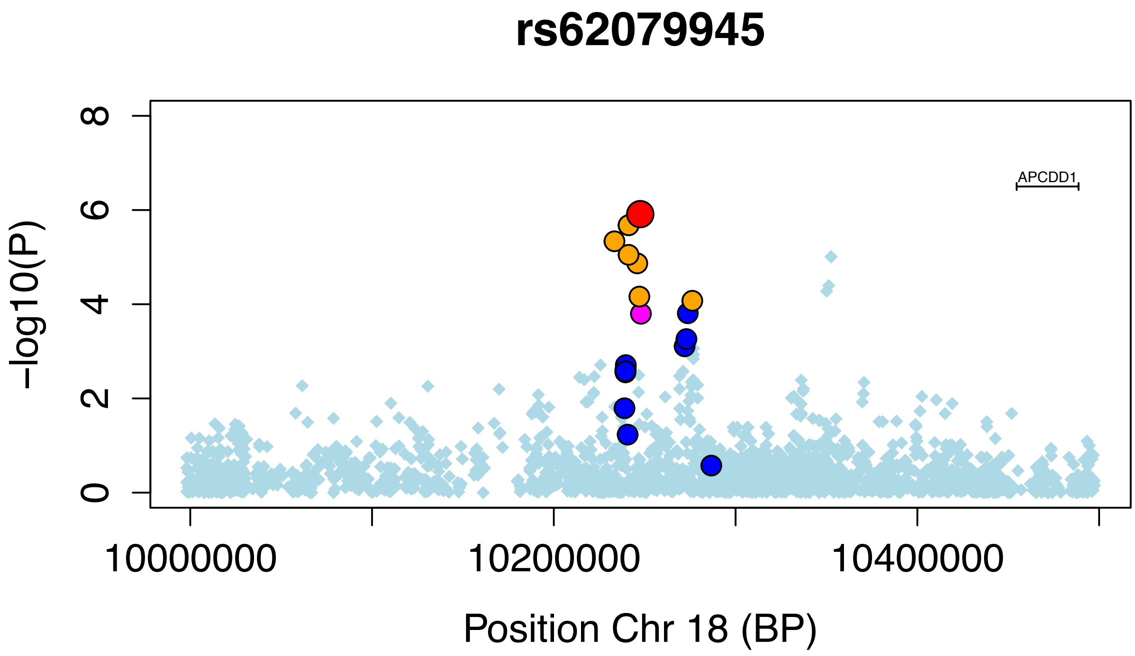
rs62079945
−log10(P)
0
2
4
6
8
APCDD1
10000000
10200000
10400000
Position Chr 18 (BP)

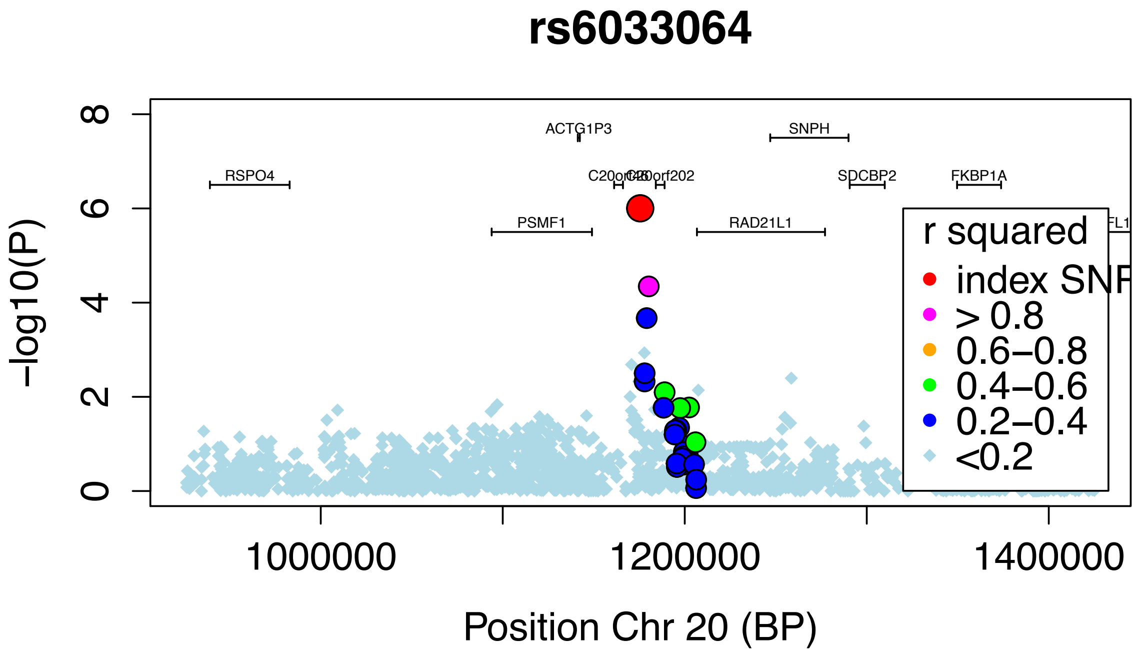
rs6033064
−log10(P)
0
2
4
6
8
ACTG1P3
SNPH
RSPO4
C20orf202
SDCBP2
FKBP1A
PSMF1
RAD21L1
r squared
index SN
> 0.8
0.6–0.8
0.4–0.6
0.2–0.4
<0.2
1000000
1200000
1400000
Position Chr 20 (BP)

Supplementary Figure 7 : Hierarchical clustering dendrogram of gene content overlaps between cross-validated pathways.

Clusters with approximate unbiased alpha values greater than 90 are marked with red polygons. Clusters filled in green had at least one pathway that was significant in each of the two pathways analyses.

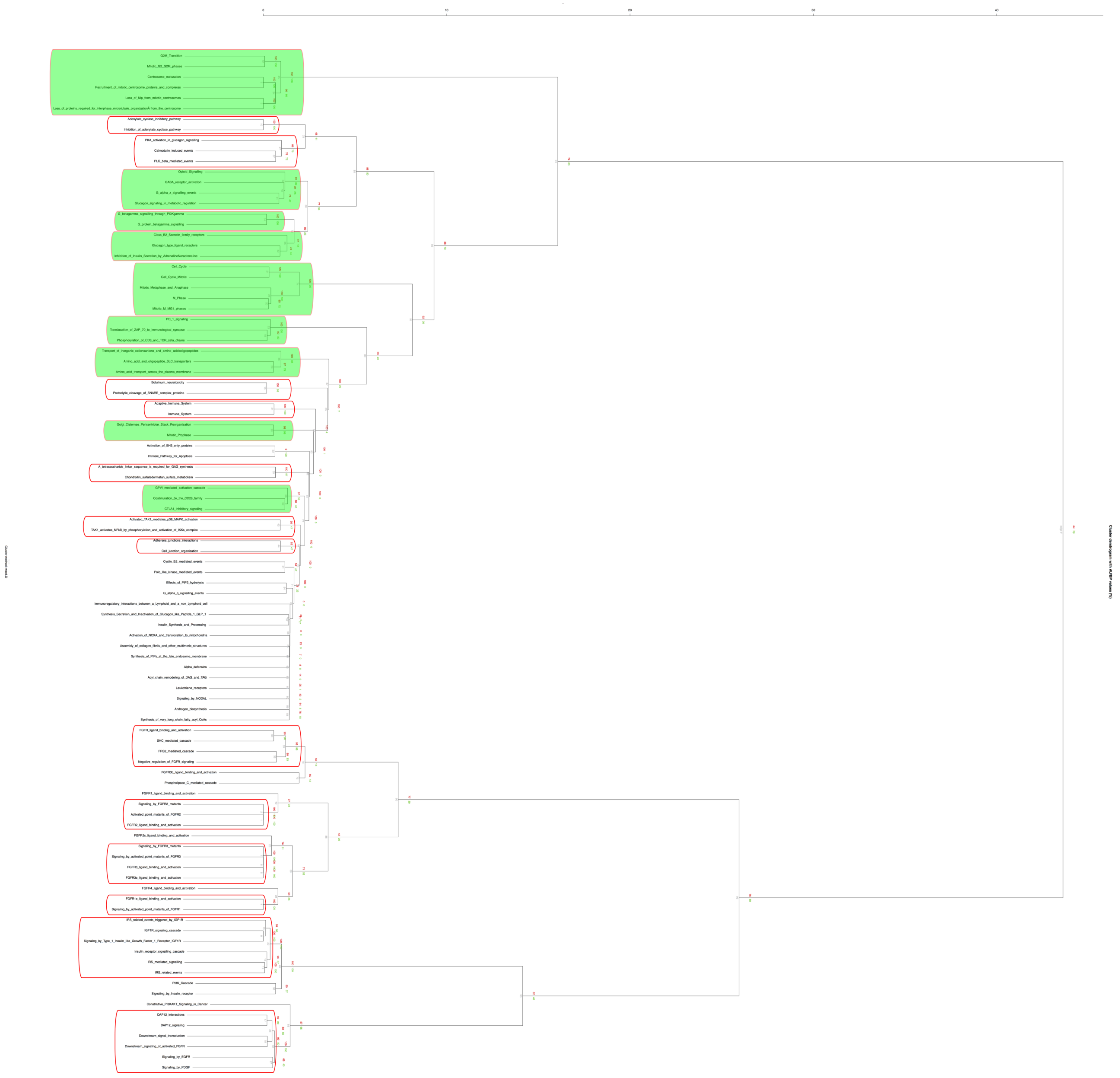
Cluster dendrogram with AU/BP values (%)
au
bp
edge #
Cluster method: ward.D
0
10
20
30
40
G2M_Transition
Mitotic_G2_G2M_phases
Centrosome_maturation
Recruitment_of_mitotic_centrosome_proteins_and_complexes
Loss_of_Nlp_from_mitotic_centrosomes
Loss_of_proteins_required_for_interphase_microtubule_organizationÂ from_the_centrosome
Adenylate_cyclase_inhibitory_pathway
Inhibition_of_adenylate_cyclase_pathway
PKA_activation_in_glucagon_signalling
Calmodulin_induced_events
PLC_beta_mediated_events
Opioid_Signalling
GABA_receptor_activation
G_alpha_z_signalling_events
Glucagon_signaling_in_metabolic_regulation
G_betagamma_signalling_through_PI3Kgamma
G_protein_betagamma_signalling
Class_B2_Secretin_family_receptors
Glucagon_type_ligand_receptors
Inhibition_of_Insulin_Secretion_by_AdrenalineNoradrenaline
Cell_Cycle
Cell_Cycle_Mitotic
Mitotic_Metaphase_and_Anaphase
M_Phase
Mitotic_M_MG1_phases
PD_1_signaling
Translocation_of_ZAP_70_to_Immunological_synapse
Phosphorylation_of_CD3_and_TCR_zeta_chains
Transport_of_inorganic_cationsanions_and_amino_acidsoligopeptides
Amino_acid_and_oligopeptide_SLC_transporters
Amino_acid_transport_across_the_plasma_membrane
Botulinum_neurotoxicity
Proteolytic_cleavage_of_SNARE_complex_proteins
Adaptive_Immune_System
Immune_System
Golgi_Cisternae_Pericentriolar_Stack_Reorganization
Mitotic_Prophase
Activation_of_BH3_only_proteins
Intrinsic_Pathway_for_Apoptosis
A_tetrasaccharide_linker_sequence_is_required_for_GAG_synthesis
Chondroitin_sulfatedermatan_sulfate_metabolism
GPVI_mediated_activation_cascade
Costimulation_by_the_CD28_family
CTLA4_inhibitory_signaling
Activated_TAK1_mediates_p38_MAPK_activation
TAK1_activates_NFkB_by_phosphorylation_and_activation_of_IKKs_complex
Adherens_junctions_interactions
Cell_junction_organization
Cyclin_B2_mediated_events
Polo_like_kinase_mediated_events
Effects_of_PIP2_hydrolysis
G_alpha_q_signalling_events
Immunoregulatory_interactions_between_a_Lymphoid_and_a_non_Lymphoid_cell
Synthesis_Secretion_and_Inactivation_of_Glucagon_like_Peptide_1_GLP_1
Insulin_Synthesis_and_Processing
Activation_of_NOXA_and_translocation_to_mitochondria
Assembly_of_collagen_fibrils_and_other_multimeric_structures
Synthesis_of_PIPs_at_the_late_endosome_membrane
Alpha_defensins
Acyl_chain_remodeling_of_DAG_and_TAG
Leukotriene_receptors
Signaling_by_NODAL
Androgen_biosynthesis
Synthesis_of_very_long_chain_fatty_acyl_CoAs
FGFR_ligand_binding_and_activation
SHC_mediated_cascade
FRS2_mediated_cascade
Negative_regulation_of_FGFR_signaling
FGFR3b_ligand_binding_and_activation
Phospholipase_C_mediated_cascade
FGFR1_ligand_binding_and_activation
Signaling_by_FGFR2_mutants
Activated_point_mutants_of_FGFR2
FGFR2_ligand_binding_and_activation
FGFR2c_ligand_binding_and_activation
Signaling_by_FGFR3_mutants
Signaling_by_activated_point_mutants_of_FGFR3
FGFR3_ligand_binding_and_activation
FGFR3c_ligand_binding_and_activation
FGFR4_ligand_binding_and_activation
FGFR1c_ligand_binding_and_activation
Signaling_by_activated_point_mutants_of_FGFR1
IRS_related_events_triggered_by_IGF1R
IGF1R_signaling_cascade
Signaling_by_Type_1_Insulin_like_Growth_Factor_1_Receptor_IGF1R
Insulin_receptor_signalling_cascade
IRS_mediated_signalling
IRS_related_events
PI3K_Cascade
Signaling_by_Insulin_receptor
Constitutive_PI3KAKT_Signaling_in_Cancer
DAP12_interactions
DAP12_signaling
Downstream_signal_transduction
Downstream_signaling_of_activated_FGFR
Signaling_by_EGFR
Signaling_by_PDGF

TABLES

Supplementary Table 1 : Complete list of SNPs with P_{EMMAX} < 5 x 10^{-6}

SNP	CHR	BP	Allele1/2 (Effect allele bold)		FREQ EA	Type	P	OR	Gene	Predicted function	Splice Dist
rs140744181	2	125615326	C	**T**	0.061	IMPUTED	4.91 x10^{-06}	0.870	CNTNAP5	INTRONIC	7557
rs58496359	3	3121314	T	**C**	0.054	IMPUTED	2.63 x10^{-6}	0.842	IL5RA	INTRONIC	3003
rs116828186	3	18854524	C	**T**	0.027	GENOTYPED	1.37 x10^{-6}	0.824			
rs28731189	5	11212170	C	**T**	0.011	IMPUTED	1.42 x10^{-6}	0.736	CTNND2	INTRONIC	12397
rs74291850	5	29577370	T	**C**	0.022	IMPUTED	4.63 x10^{-7}	1.291			
rs187984259	5	73681906	T	**C**	0.016	IMPUTED	1.43 x10^{-6}	0.758			
rs145595245	6	124070632	C	**A**	0.020	IMPUTED	2.07 x10^{-6}	1.313			
rs80317841	6	132398394	**G**	C	0.840	GENOTYPED	1.97 x10^{-6}	1.092			
rs73257509	6	165574984	C	**T**	0.167	IMPUTED	3.76 x10^{-6}	0.917			
rs116141145	7	97249265	A	**G**	0.037	IMPUTED	1.24 x10^{-6}	1.190			
rs114060470	7	97260121	G	**A**	0.036	IMPUTED	1.37 x10^{-6}	1.205			
rs1403864	7	118237253	**A**	C	0.624	IMPUTED	4.66 x10^{-6}	1.075			
rs111513399	8	69036056	A	**S**	0.113	IMPUTED	5.38 x10^{-7}	1.114	PREX2	INTRONIC	2772
rs10809114	9	10532173	C	**T**	0.064	IMPUTED	2.88 x10^{-6}	0.880	PTPRD	INTRONIC	80225
rs11258313	10	13339766	G	**A**	0.233	IMPUTED	1.35 x10^{-6}	1.101	PHYH	INTRONIC	421
rs201134023	10	18922338	-	**ACA**	0.216	IMPUTED	1.72 x10^{-6}	1.081	NSUN6	INTRONIC	9067
rs11614525	12	97692730	A	**T**	0.042	IMPUTED	2.70 x10^{-6}	1.190			
rs2173866	12	101950920	**C**	G	0.716	IMPUTED	3.60 x10^{-6}	0.932			
rs116221519	13	22312484	G	**A**	0.038	IMPUTED	3.73 x10^{-6}	1.191			137271
rs34333926	13	94017140	C	**T**	0.011	IMPUTED	2.74 x10^{-6}	0.677	GPC6	INTRONIC	
rs142646255	15	58034533	GAG	**-**	0.031	IMPUTED	3.91 x10^{-6}	0.811			4136
rs1531683	15	84679158	**T**	C	0.711	GENOTYPED	4.23 x10^{-6}	0.934	ADAMTSL3	INTRONIC	1536
rs9895748	17	5053598	T	**A**	0.068	IMPUTED	1.72 x10^{-6}	0.872	USP6	INTRONIC	
rs62079945	18	10247566	A	**C**	0.048	IMPUTED	1.22 x10^{-6}	0.842			
rs6033064	20	1175527	**T**	C	0.803	IMPUTED	9.94 x10^{-7}	0.909			
rs1487321	20	44235865	T	**G**	0.391	IMPUTED	3.38 x10^{-6}	1.070	WFDC9	3′ DOWNSTREAM	
rs147707056	21	16582162	A	**T**	0.014	IMPUTED	3.19 x10^{-6}	0.726			

*Expected frequency of the effect allele
**The OR indicates the estimated allele frequency odds ratio for the effect allele. Values less than one indicate that the effect allele is less common in cases than controls and vice versa.

Supplementary Table 2 : Potential roles for GWAS tagged candidate genes in Chlamydia mediated pathology

Gene	Supporting Evidence	Potential Role in Ct pathology	REF
PREX2	• GNEFs Sos1 and Vav2 interact with Chlamydial TARP, PI3K and Rac<br>• PREX2 has similarity to Sos1 and Vav2 and is known to interact with Rac and the PI3K inhibitor PTEN	• TARP mediated Ct entry<br>• Additional role for PREX2 in glucose homeostasis	1<br>2
PHYH	• PHYH oxidises branched-chain fatty acids in the peroxisome	• Limiting lipid supply to inclusion	3–6
USP6	• NF-κB activation<br>• ERK, MAPK and MyD88 signalling. Control of cell motility and cytokinesis	• NF-κB activation, stimulation of MMP9<br>• Control of cell cycle arrest and limiting cytokinesis	7–9<br>10
CTNND2	• Another catenin (beta) is involved in cell cycle regulation and has been shown to be sequestered to the cytoplasmic inclusion during infection<br>• Beta catenin involved in Wnt signalling	• Disruption of cell-cell junctions<br>• Cell cycle arrest	11<br>12
NSUN6	• A paralogue of the *NOP2* gene and a regulator of cell proliferation	• Cell cycle arrest	13

Supplementary Table 3 : Potential roles for pathways-wide GO terms in Chlamydia mediated pathology

GO	Supporting Evidence	Potential Role in Ct pathology	REF
Microtubule-based Process	• Ct causes mitotic spindle pole defects<br>• Association of Ct with the centrosome	• Establishment of the parasitic niche	14,15
G-protein coupled Receptor protein signalling pathway	• Involvement of GNEFs in TARP mediated cellular entry by the elementary body	• Initiation of invasion, cytoskeletal reorganisation and downstream cell cycle control<br>• Association with C. muridarum infection related upper genital tract disease severity	1<br>16
Cellular response to hormone stimulus	• Close relationship to G-protein signalling<br>• Hormone receptors shown to aid chlamydial entry<br>• *C. pneumoniae* protein Cpn0712 down-regulates glucagon-Like-Peptide Receptor 2<br>• Direct roles for Glucagon-like-Peptide 1 in fibrosis	• Enhance binding and entry of Ct, potentially via GLP1R or ILGFR<br>• Link to PI3K/Akt/p53, apoptosis and glucose homeostasis<br>• Possible pathway towards scarring	17–20
Cell surface receptor linked signal transduction	• GNEFs in TARP mediated cellular<br>• FGF receptors directly bind EBs during infection<br>• FGF2 potentially acts as a bridging molecule to directly facilitate the binding of elementary bodies	• Downstream signalling targets cell cycle<br>• Increased binding to host cells during the initial phase of infection	1,21
Cell Cycle	• p53 degradation releases G6PD activity. Diversion of glycolysis substrates to the pentose phosphate pathway.<br>• Ct derived CPAF acts as an anaphase promoting factor by cleaving cyclin B2 and securin<br>• CPAF degrades p53<br>• miR-1285, upregulated in TS, inhibits p53<br>• Ct modifies cyclin dependent kinase (CDK) activity<br>• Ct disrupts cytokinesis, overrides mitotic spindle checkpoint and induces early progression through mitosis.	• Enhanced energy, nucleotide and amino acid harvesting to inclusion from cytoplasm<br>• Cell cycle control<br>• control of G6PD, cell cycle and NF-κB<br>• CDK family members are implicated in fibrotic responses<br>•	22–28,15
Regulation of T cell activation	• CD4 and CD8 T cells involved in both protection and pathology	• IFN-G response leading to pathology	reviewed [29]
Sodium Ion Transport	• Ct manipulates Na^+ homeostasis leading to cytoplNasmic pH change and cell cycle changes	• May mediate appropriate energy and metabolism control in reticulate bodies	reviewed [30]
Phosphorous metabolic process	• Non-significant GO term<br>• This term is a proxy for prophase and golgi stack organisation	• Ct disrupts the golgi apparatus and causes golgi ministacks to form in proximity to inclusion, aiding reproduction	31
Regulation of phosphorylation	• Includes pathways of T cell immunity and extracellular matrix and platelet activation	• CD24, CTLA4, Glycoprotein IV<br>• Scarring and immune response	

Supplementary Table 4. Summary statistics for ALIGATOR analysis.

The number of significant pathways at p ≦ 0.01 and p ≦ 0.05, given 1,345 pathways and cutoff values of either 0.01, 0.001 or 0.0001 are given.

		Threshold value*			
		0.01	0.001	0.0001	Expected**
Number of significant pathways	P ≦ 0.01	9	51	18	13.45
	P ≦ 0.05	50	103	39	67.25

* Threshold value refers to ALIGATOR snp.pcut argument in R SNPath package

** number of pathways expected to return the specified p value by chance alone, after 100 permutations and given 1345 possible pathways

METHODS

Tests for population stratification

The directly genotyped SNP data and the 1000 genomes reference data [31] were filtered to obtain a subset of variants with MAF ≥ 0.01 and HWE p-value > $1x10^{-5}$ in both datasets. The remaining SNPs were then pruned for LD independence in the dataset with a sliding window of 500 SNPs and a maximum r^2 LD value of 0.2. The final set of SNPs was used to calculate ethnic ancestry Principle Components Analysis axis first on a global scale using all 1000 genomes populations and subsequently on an a continental scale using only populations with predominantly African ancestry. Results of this analysis can be seen in supplementary figures 3 and 4.

Pathways analysis

ALIGATOR counts the number of genes in a pathway that contain a SNP with a P_{EMMAX} value more extreme than a pre-specified threshold value and then determines the significance of pathways in permutation tests. The signal to noise ratio of the test can be controlled by calibration of the P threshold value that is considered nominally significant. The ALIGATOR literature recommends exploring the effects of utilising a number of different thresholds. Threshold values that return a greater number of significant pathways than the number of pathways expected to be significant by chance alone at $P < 0.01$ and $P < 0.05$ is predictable (given 1345 pathways) as respectively 13.45 and 67.25. An optimal threshold for pathways discovery might be one that returns more significant pathways than would be expected by chance alone. In this study, the effects of using threshold values of 0.01, 0.001 and 0.0001 were explored. Using a threshold value of 0.01 led to fewer significant pathways than would be expected by chance (Supplementary table 1), suggesting that this cut-off was too relaxed. Threshold values of 0.001 and 0.0001 both obtained more significant pathways than expected,

with a peak at 0.001 (Supplementary table 1), which was then used in the main analysis.

The high number of SNPs involved, combined with a high number of permutations, makes this process computationally intensive and a relatively modest number (n = 100) of re-samplings were initially used to pre-screen the pathways. Candidate pathways for fine ALIGATOR analysis were those with $p < 0.05$ in the initial screening. Candidate pathways were then tested again by ALIGATOR using 100,000 permutations of the phenotypes.

For each pathway in PODA analysis, a score "S" is determined for each individual's sample. This score describes the genetic distance of the current sample from other cases relative to its distance from controls. The distributions of these scores in cases and controls are then compared to obtain the pathway Distinction Score 'DS'. DS is normalised by resampling with randomisation of the phenotypes and is tested for significance by an implementation of the permutation test where a set of arbitrary pathways of equal length to the pathway of interest are tested for association with the phenotype with resampling of phenotypes. The DSp is a confidence measure that is analogous to a standard p-value and that describes the proportion of permutations in which the DS value was larger in the simulated pathway than in the true pathway. The reported Odds Ratio (OR) for the pathway describes the increase in relative odds of disease given each unit increase in S. For each pathway we implemented 100 re-samplings and 1000 permutations of the test.
